# A fuzzy system based self-adaptive memetic algorithm using population diversity control for evolutionary multi-objective optimization

**DOI:** 10.1038/s41598-025-89289-2

**Published:** 2025-02-17

**Authors:** Brindha Subburaj, S. Miruna Joe Amali

**Affiliations:** 1 School of Computer Science and Engineering, Vellore Institute of Technology, Chennai, Tamilnadu India; 2 Department of Computer Science and Engineering, K.L.N. College of Engineering, Sivagangai, Tamilnadu India

**Keywords:** Evolutionary computations, Multi-objective optimization, Fuzzy system, Population diversity, Memetic algorithm, Engineering, Computer science

## Abstract

Simulated by nature’s evolution, numerous evolutionary algorithms had been proposed. These algorithms perform better for a particular problem domain and extensive parameter fine tuning and adaptations are required in optimizing problems of varied domain. This paper aims to develop robust and self-adaptive memetic algorithm by combining Differential Evolution based algorithm, a popular population based global search method with the Controlled Local search procedure to solve multi-objective optimization problems. Memetic Algorithm is an enhanced evolutionary algorithm, it combines global search method with local search techniques for faster convergence. Memetic algorithm improves both exploration and exploitation, preventing premature convergence and also refines the current best solutions efficiently. Proposed algorithm is named as Fuzzy based Memetic Algorithm using Diversity control (F-MAD). In F-MAD, population diversity is controlled through the control parameters self-adaptation of Differential Evolution algorithm (DE) such as, crossover rate and scaling factor by using two fuzzy systems. A controlled local search procedure is adapted for guiding convergence process thus balancing explore-exploit cycle. The control parameter self-adaptation and enhanced selection method with controlled local search method aid population diversity control in decision space and attaining optimal solutions with uniform distribution in terms of diversity and convergence metrics in objective space. These characteristics help the proposed method suitable to be extended to different application domain without the need of trial-and-error fine tuning of the parameters. The performance is tested through standard benchmark test problems-CEC 2009 test problems and DTLZ test problem and further validated through performance metrics and statistical test. It is compared with popular optimization algorithms and experiment results indicate that F-MAD perform well than State of-The-Art (SOTA) algorithms taken for comparison. F-MAD algorithm attains better results for 8 out of 10 CEC 2009 test problems (UF1-UF10) when compared to 20 other algorithms taken for comparison. For DTLZ problems, F-MAD attains better results for ALL 7 problems (DTLZ 1-DTLZ7) when compared to 8 other SOTA algorithms. The performance is further evaluated using Friedman rank test and the proposed F-MAD significantly outperformed other algorithms.

## Introduction

Science and engineering problems in real world includes multiple conflicting objectives required to be optimized and are called multi-objective optimization Problems (MooPs). The aim is to minimize or maximize the values of objective function. Since the objectives are conflicting, the solutions are multiple trade-offs namely Pareto optimal solutions. Multi-objective optimization deals with optimizing multiple conflicting objectives simultaneously. Instead of a single population best solution, MooP produces a set of solutions, called Pareto optimal solutions. The objective in such MooP is to find a well-distributed Pareto front, which is a good trade-off between objectives.

Evolutionary Algorithms (EA) are popular and widely adapted approach to solve optimization problems. EA’s mimic nature’s evolution process and are population of solutions based search and optimize algorithm. “Elitist Non-dominated Sorting Genetic Algorithm (NSGA-II)”^1^, “Strength Pareto Evolutionary Algorithm (SPEA2)”^2^, “Multi-objective Genetic Algorithm (MOGA)”^3^ are the benchmark and classic multi-objective evolutionary algorithms.

“Differential Evolution (DE)”^4^, a popular class of EA and showed its efficiency in solving complex optimization problems which is evident from earlier research articles in this field. Several representative algorithms have been proposed based on DE approach^5^. In “multi-objective optimization based on Self-adaptive Differential Evolution (MosaDE)”^6^, authors improved a single-objective DE based optimization method using self-adaptation^7^ suitable for solving MooPs. Trial vector strategy pool and its self adaptation, control parameter adaptation is proposed. Evaluation of potential solution by considering dominance factor and harmonic distance measure is suggested and their results show capability of Self-adaptive Differential Evolution in solving MooPs. In^8^ authors proposed “Multi-objective Differential Evolution Algorithm (MODEA)” by improving initialization and mutation techniques of DE using random localization and opposition-based learning concepts. Selection is based on comparing target and trial vectors and further non-domination based selection is suggested.

Optimization techniques by controlling the elitism factor is proposed in $\:\alpha\:$-DEMO-revised algorithm through a pre specified proportion^9^. In^10^ authors have proposed $\:pa\epsilon$-ODEMO which is an improved multi-objective DE based optimization algorithm. In $\:pa\epsilon$-ODEMO the initial population is generated using orthogonal design technique by maintaining uniformity among the solution and an enhanced $\:pa\epsilon$-dominance technique is used, these changes improves the convergence and diversity properties. Decomposition technique based optimization algorithms for solving multi-objective problems are widely used and one such is “MOEA/D, A multiobjective Evolutionary algorithm based on Decomposition”^11^ where problem with multiple objectives is split in to group of sub problems using Tchebycheff method. The sub problems are solved simultaneously with the neighbourhood sub problem information. One of the several methods proposed by improving the MOEA/D algorithm is “External archive matching strategy for MOEA/D (MOEA/D-EAM)” algorithm where external archive is used to save nondominated solutions which are used for reproduction in later evolution stages thus improving the convergence of the algorithm^12^.

The above-mentioned optimization approaches fall under the global search category and the methods which solves optimization problems by performing search in promising regions are called Local Search (LS) method. LS methods work by evaluating the points surrounding a random solution to find a better solution than current random solution and if one such is identified it is retained. LS methods though exhibit a faster convergence rate due to the above method of searching, there are few issues to be considered while extending it. A major concern of LS method is the loss of lateral diversity along the Pareto optimal fronts as an effect of excessive exploitation.

In^13^ authors proposed NSLS – “Nondominated Sorting and Local Search based algorithm” where after population initialization, an improved population is derived by using a local search method. Later both the initial and improved populations are united. Nondominated sorting is performed over the combined population assisted with farthest-candidate approach to obtain better solutions. In the “Local search based Evolutionary multi-objective optimization algorithm”^14^, NSGA-II algorithm is used for offspring generation and later LS is applied to generate improved offspring where, a saw-tooth function is used to control over exploitation. The parent and improved offspring solutions are united and further a non-dominated sort method assisted with k-means clustering technique is extended to derive new population to continue with search process and the lateral diversity loss is prevented by this strategy. Few other Local search based algorithms^15,16^ have been widely applied to solve a domain specific optimization problems.

Memetic Algorithm (MA) based on meme and natural evolution is a hybrid method combining global search with local refinement capability^17^ and many algorithms had been proposed in recent times and the algorithms exhibit fast convergence measure with respect to pareto optimal solutions through higher precision. In^18^ authors proposed MOEA/D-GLS, which is a multi-objective memetic algorithm by combining decomposition based EA and guided local search method through which the exploiting ability of decomposition based MOEA’s is promoted. In Adaptive memetic computing^19^ synergetic combination of multiple evolutionary algorithms is proposed and implemented in domination and decomposition-based algorithms and showed the efficiency in solving MooPs. In the “Opposition-based Self-adaptive Hybridized Differential Evolution (OSaDE)” method^20^, Global search variant DE algorithm is combined with gradient search method and succeeded in reaching optimal solution, balancing explore-exploit cycle. Further in several other research articles^21–24^, DE is combined with a local search method and their results are evident to further proceed research in the direction.

Recent multi-objective evolutionary algorithms developed and applied to solve specific application problems are reviewed and presented. In^25^ a 2-Archive Multi-objective Cuckoo Search (MOCS2arc) is developed to solve the structure optimization problem, where dual archive is used to improve diversity of the solutions. In^26^, authors have used 13 meta heuristics algorithms to optimize UAV system identification. L-SHADE algorithm is reported to have better results through statistical test results. In^27^ a memetic technique using sine cosine trigonometric function based optimizer and a $\:\beta\:$-hill climbing local searh technique is used to solve the economic load dispatch problem and the experimentation results are better or at par for the most testcases. A hybrid artificial bee colony algorithm is proposed to solve the nurse rostering problem^28^. The exploitation capability is improved in this algorithm using a hill climbing optimizer, and competitive results are reported.

In^29^ the multi-objective optimization problem related to micro-grid power dispatch using a penalty based boundary intersection and elite learning technique is proposed. The objectives of attaining minimum economic cost and balancing the other objectives voltage and frequency stability is achieved through the demonstrated algorithm. In^30^, type-3 fuzzy system is used for the path tracking task of autonomous cars. The results reported proves the robustness of the fuzzy system in adapting and solving such uncertain real-world tasks. The current research work is a fuzzy system based control parameter adaptation. In^31^ NSGA-II algorithm is used to solve the multi-objective optimization problem related to opportunistic maintenance. Where, the objectives is to maximize operational reliability and to minimize maintenenace cost. The attained results are demonstrated to be better than traditional model results. In^32^ Bayesian optimization algorithm is used to optimize the escape entrapment control strategy related to the planetary rover. The rover’s trajectory using the above technique based gait is reported to be accurate.

The focus of the article is to develop a memetic algorithm by enhancing DE algorithm and combining with a controlled local search procedure. DE, a population-based search and optimize algorithm can succeed only if the population diversity is high else, the algorithm may face population stagnation or premature convergence. The aim is to improve diversity among population through control parameter self-adaptation using Fuzzy System (FS) and combining it with a controlled local search procedure thus balancing exploration and exploitation cycle, making the algorithm robust and appropriate to extend for varied problem domain without the need to manually fine tune the control parameters.

There are two search spaces involved in multi-objective optimization namely decision and the objective space. Searching for optimal solutions is performed at decision space and the diversity among the solutions is a crucial factor to help in searching diverse region in the search space. Whereas, the objective space is a representation of various objective functions involved in multi-objective optimization and are multi-dimensional. The goal of any multi-objective optimization algorithm is to reach solutions that approximates well with the Pareto optimal front and is assessed using two factors convergence of the obtained front towards the true Pareto front and the diversity aspect along the attained solutions. Diversity of solutions in both spaces is required to identify global optima. Appropriate techniques like population diversity based adaptation of the control parameters and enhanced non-domination based selection methods with a controlled local search procedure are implemented to balance and improve population diversity in decision space and diversity and convergence measures towards True Front in objective space.

The major contributions include:

1. Extending fuzzy system for self-adapting crossover and scaling factor values associated with DE based algorithm.
2. Controlled local search procedure to improve exploitation and the modification of selection procedure of DE algorithm by using improved non-domination method.
3. New multi-objective Memetic algorithm by combining the Differential Evolution based algorithm, a popular population based global search method with the proposed Controlled Local search procedure.
4. Experimenting the performance of the proposed algorithm in optimizing benchmark test problems, evaluated using quality indicator and through non-parametric statistical test.

The proposed method is an enhancement to “Fuzzy Adaptive Multi-objective Differential Evolution with Diversity Control (FAMDE-DC)” algorithm^33^ by extending two fuzzy systems to adapt crossover and scaling factor and introducing a controlled local search procedure. These techniques improve the robustness of the presented F-MAD algorithm which are evident through the attained results.

The proposed F-MAD algorithm adapts the trial vector generation strategy and the control parameters crossover rate and scaling factor using fuzzy system based on the current population diversity and based on objective function value of current solutions in the population compared with best individual, the fine tuning of crossover rate and scaling factor values based on this information better handles the search process with improved diversity measures as required. To improve the exploitation, controlled local search technique is proposed, thus making the algorithm robust enough in handling varied problem types through its self-adaptation capabilities according to the search space.

The paper is organized such as, Sect. 2 details the proposed algorithm. In Sect. 3, experimental setup and performance of algorithm is analysed on different benchmark test problems, Sect. 4 is application of the proposed algorithm to solve a real world optimization problem and its results Sect. 5 gives the conclusions.

## Proposed multi-objective F-MAD algorithm

The proposed F-MAD algorithm is detailed in this section. Evolutionary algorithms perform well while there is diversification in population. Loss of population diversity leads to premature convergence and stagnation. Control parameters like crossover rate and scaling factor decide the performance of Differential evolution based optimization algorithms. A Robust multi-objective memetic algorithm namely Fuzzy based Memetic Algorithm using Diversity control (F-MAD) is proposed in which, population based global search algorithm namely Differential Evolution is combined with a controlled local search procedure. In F-MAD, techniques to self- adapt the control parameters using fuzzy system based on population diversity are proposed and selecting potential individuals using fast non-dominated sorting method by controlling the level of elite solutions entering next generation and using dynamic crowding distance metrics^34^. To improve convergence properties a controlled local search procedure is proposed thus, balancing the explore-exploit cycle.

**Fig. 1:**
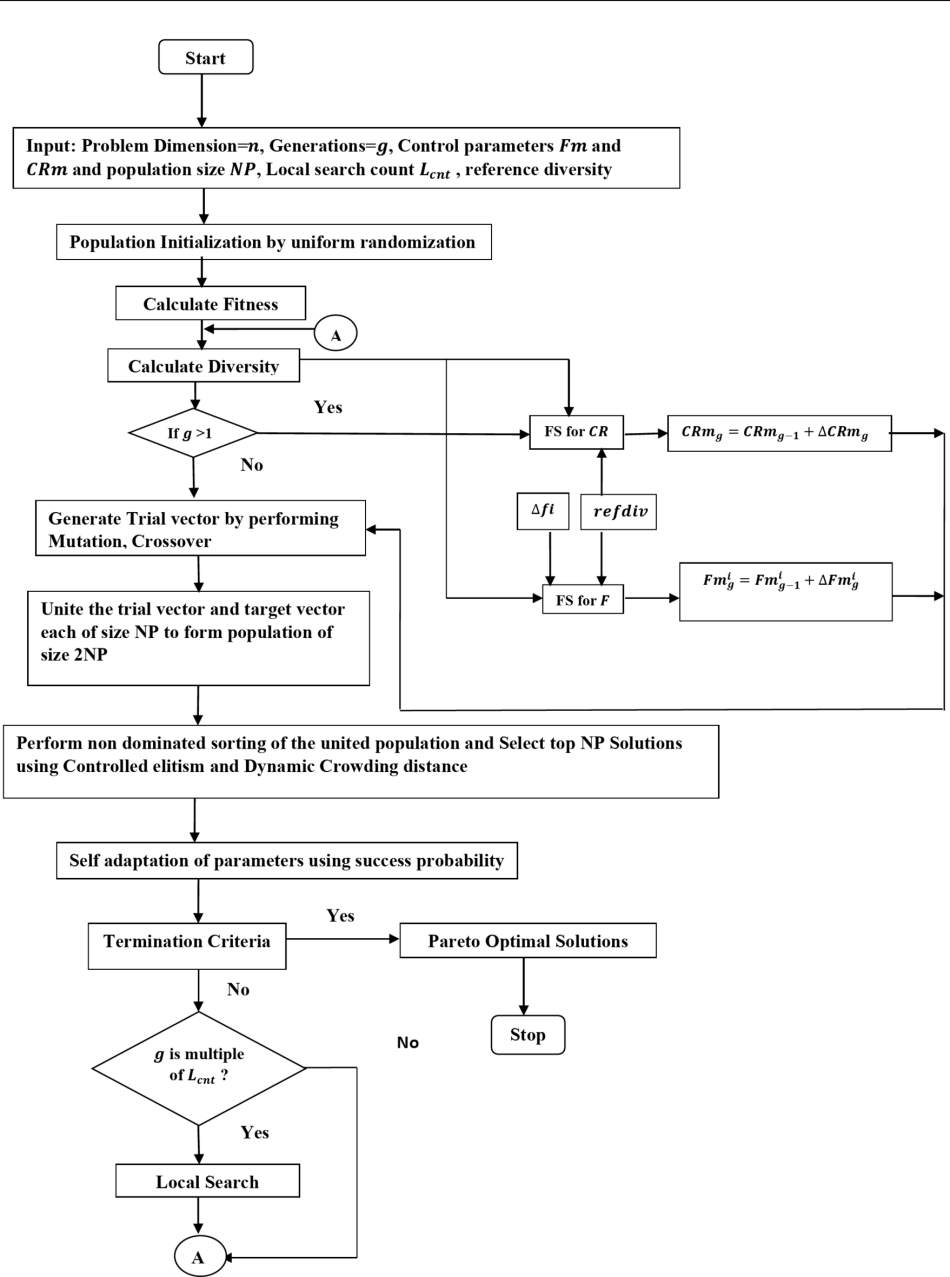
Flowchart of F-MAD algorithm.

The explore-exploit cycle in evolutionary algorithms balances diversity and refinement. Exploration helps discover new regions by introducing variation through operations like mutation and crossover), preventing premature convergence. On the other hand, Exploitation focuses on improving the current best solutions using techniques like local search. Early generations is more towards having better exploration, while later stages move towards exploitation for fine-tuning. A well-balanced explore-exploit cycle ensures both global search (exploration) and local optimization (exploitation), leading to optimal solutions. The Control parameter self-adaptation and enhanced selection method improves diversity in both decision and objective space further, combining it with local search procedure results in identifying potential solutions exhibiting good performance measures. The steps in F-MAD algorithm are illustrated in Fig. 1 and through the below algorithm.

**Figure:**
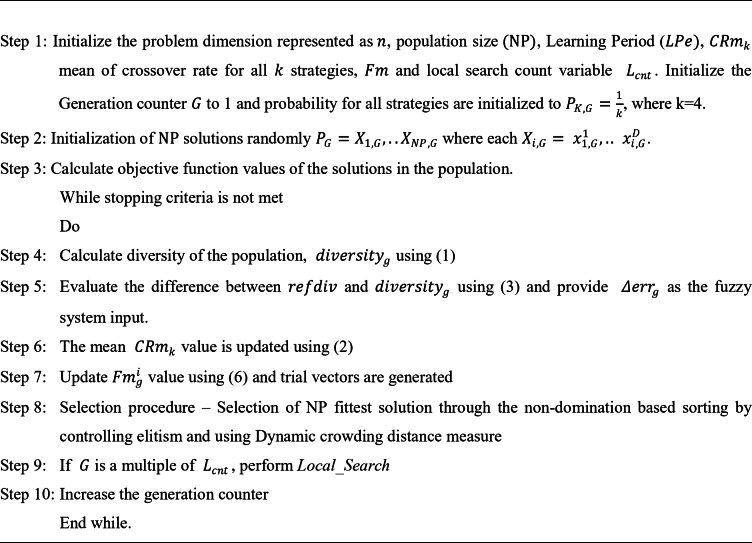
Multi-objective F-MAD algorithm.

Initializing population is the primary task in EA. Search and optimize methods proceed with initial set of solution and attempt to improve them. In this work initial population of solutions are generated randomly constrained on variable bounds.

After initialization trial vectors generated using trial vector generation strategy. The performance of each strategy varies across different problem domains. Strategies behaviour also differs during various stages of evolution. Thus identifying a particular strategy to solve varied optimization problems is not feasible. Thus strategy pool^35^ is maintained with four trial vector generation strategies strategy 1-DE/rand/1/bin, strategy 2 - DE/rand-to-best/2/bin, strategy 3- DE/rand/2/bin and strategy 4- DE/current-to-rand/1. Among which the method to choose population best solution in the strategy “DE/rand-to-best/2/bin” is modified according to MooPs and is selected randomly from first front obtained by fast non-dominated sorting. The Objective function values are estimated for all the solutions in the population and they are subject to non-dominated sorting and categorized in to fronts and a random solution from the first and best non-dominated front is selected as current population best solution for the generation. Trial vectors are generated using the above pool of four trial vector generation strategies.

A particular trial vector generation strategy from the pool of strategies is chosen using a success index value of that particular strategy, which indicates how far the strategy is successful in generating potential solutions in yester iterations. If there are $\:K$ strategies in the strategy candidate pool, then the probability of selecting $\:{k}^{th}$ strategy for trial vector generation is represented as $\:{P}_{k}$, where $\:k=\text{1,2},.K.$ All the considered strategies are given equal probability of $\:\frac{1}{k}$ during initial Learning Period ($\:LPe)$. After$\:\:LPe$, the strategy adaptation is initiated and the probability of choosing a strategy is updated by tracking success and failure index of a particular strategy. The probability of using a $\:{k}^{th}$ strategy for trial vector generation is high if they produce promising solutions during the learning period and in previous generations. This method of strategy adaptation improves the search performance at different evolution periods and makes the algorithm robust in handling problems exhibiting distinct characteristics.

### Fuzzy system based control parameter adaptation

The fuzzy system developed to adapt the crossover rate and scaling factor is detailed in this section. For the DE based algorithms, following are the vital control parameters: size of population $\:\left(NP\right)$, crossover rate $\:\left(CR\right)$ and scaling factor $\:\left(F\right)$. $\:NP$ is defined by the user according to problem complexity. Trial vectors are generated through the previously mentioned strategies and their performance is based on values of control parameters $\:CR$ and $\:F$. Appropriate $\:CR$ and $\:F$ values improve the search efficiency and convergence towards optimal solutions. EA performance is correlated to population diversity, highly diversified population leads to search wider region in search space thus help escape from population stagnation and premature convergence. $\:CR$ and $\:F$ are the parameters that aid in searching diverse regions. Manual fine tuning of $\:CR$ and $\:F$ values based on trial-and-error method specific to a particular problem will not help in identifying optimal solutions for other problems. Thus, a fuzzy based method to self-adapt $\:CR$ and $\:F$ based on population diversity is used to solve MooPs.

In the earlier work, single FS is used for adapting the crossover rate value^33,36^. In this work it is further enhanced and Two Fuzzy Systems (FS) are used to adapt $\:CR$ and $\:F$ values. FS improve the mean of normal distribution used for generating $\:CR$ and $\:F$ values, by considering population diversity. First population diversity is found and then difference from reference diversity is estimated and given as input to FS and the system outputs the improved crossover rate mean $\:CRm$ and scaling factor mean $\:Fm$. These mean values are later used for crossover rate and scaling factor values generation for the current generation with ability to search better.

‘Distance to average point’ metric is used for calculating population diversity^37^ using:

1 $$\:{diversity}_{g}\left(P\right)=\:\frac{1}{\left|LD\right|\text{*}NP}\text{*}{\sum\:}_{i=1}^{NP}\sqrt{{\sum\:}_{j=1}^{{D}_{i}}({x}_{ij}-{\overline{x}}_{j}}{)}^{2}$$

Where, variable $\:NP$ denotes the population size, search space diagonal length is denoted by $\:\left|LD\right|$ estimated using $\:\sqrt{\sum\:({x}_{max}-{x}_{min}{)}^{2}}$. Problem dimension is denoted as $\:{D}_{i}$ and $\:{x}_{ij}$ is the j^th^ value of i^th^ individual and $\:{\overline{x}}_{j}$ is the j^th^ value of search region’s average point $\:\overline{x}$.

**Fig. 2:**
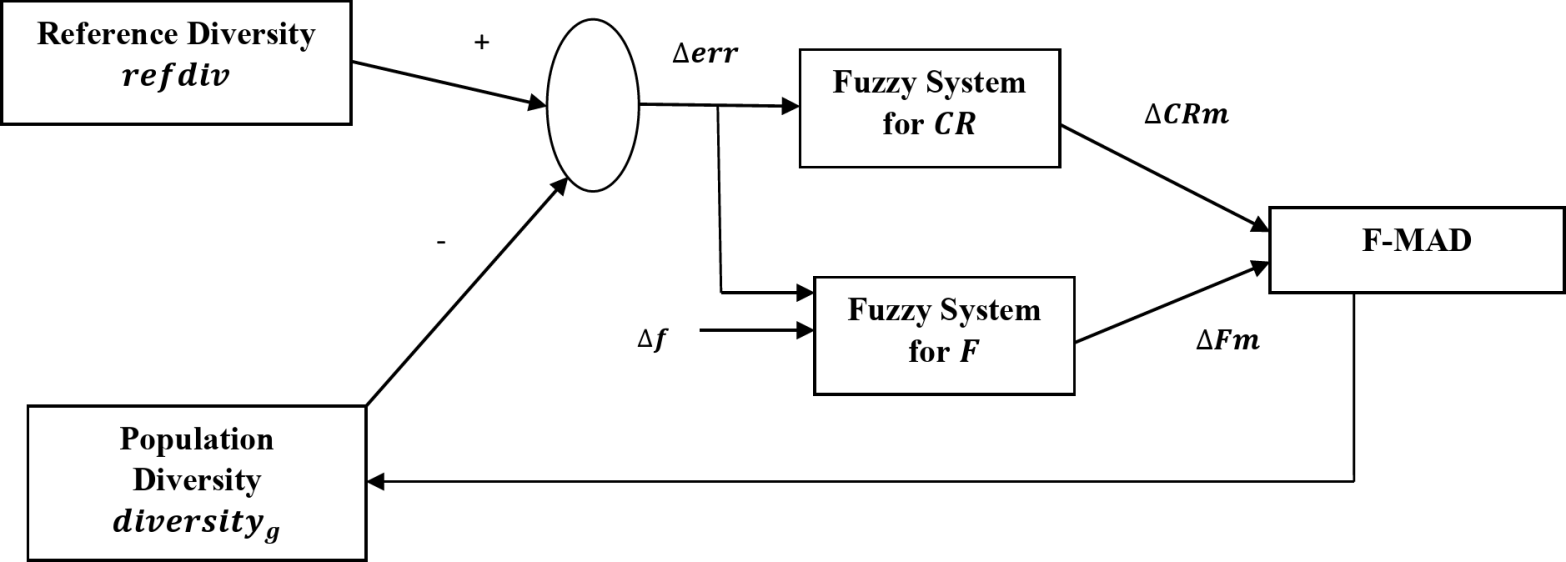
Control Parameters Crossover rate and Scaling factor adaptation using Fuzzy System.

Mamdani’s model of fuzzy system^38^, is extended to construct FS to self-adapt $\:CRm$ and $\:{F}_{m}$. The FS is illustrated in Fig. 2.

#### Crossover rate adaptation using fuzzy system

The fuzzy system designed to adapt crossover rate is presented in this section. Crossover rate ($$\:\text{C}\text{R})$$ used in mutation (trial vector generation strategy) is calculated based on normal distribution $$\:N\left({CRm}_{k},std\right).$$$$\:{CRm}_{k}$$ is the crossover rate mean with respect to $$\:{k}^{th}$$ strategy and $$\:std$$ is the standard deviation value set at 0.1. The Initial $$\:{CRm}_{k}$$ is set as 0.5 for all $$\:k$$ strategies and $$\:\text{C}\text{R}$$ values produced through normal distribution $$\:N({CRm}_{k},0.1)$$ and these $$\:\text{C}\text{R}$$ values are applied to respective $$\:{k}^{th}$$ strategy to generate target vector. After learning period ($$\:LPe)$$ generations, $$\:{CRm}_{k}$$ is generated using two parameters:

2 $$\:{CRm}_{k}={CRm}_{s,k}+{CRm}_{d,k}$$

Where $\:{CRm}_{s,k}$ value is derived as indicated in SaDE algorithm. It is the median value derived from $\:\text{C}\text{R}$ values with respect to $\:{k}^{th}$ strategy which are successful in generating promising solutions in the learning period ($\:LPe)$. The value $\:{CRm}_{d,k}$ is obtained using fuzzy system which is based on population diversity.

Single input/output fuzzy system is used to self-adapt the crossover rate mean based on the current population diversity for solving MooPs. At each generation, the current population diversity based on target vector is calculated using (1) represented as $\:{diversity}_{g}$, a user defined reference diversity ($\:refdiv$) is initialized and the difference between $\:{diversity}_{g}$ and $\:refdiv$ is calculated to find the deviation level of current population diversity from an expected level and the difference is termed as diversity error ($\:{\varDelta\:err}_{g}$)

3 $$\:{\varDelta\:err}_{g}=refdiv-\:{diversity}_{g}$$

Where $\:{\varDelta\:err}_{g}$ is the fuzzy input and fuzzy system generates$\:{\:\varDelta\:CRm}_{k}^{g}$, the needed change in crossover rate mean with respect to $\:{k}^{th}$ strategy to improve population diversity. Linguistic fuzzy variables to represent input and ouput to fuzzy system are Zero Difference (ZD), Negative Difference (ND), Positive Difference (PD), Largely Negative Difference (LND), Largely Positive Difference (LPD). Triangular membership function is used to define fuzzy variables. Defuzzification is done based on centroid technique. The implication rule base for crossover rate adaptation FS is listed in Table 1.

**Table 1:** Fuzzy rules for adapting crossover rate.

*Δerr*	LND	ND	ZD	PD	LPD
*ΔCRm*	LND	ND	ZD	PD	LPD

The above rule set is framed such as, for instance if $\:\varDelta\:err$ is Positive Difference (PD) which implies that current population diversity $\:{diversity}_{g}$ is less than the reference diversity and to improve population diversity, crossover rate has to be increased thus $\:\varDelta\:CRm$ value is Positive Difference. Crossover rate mean for $\:{k}^{th}$ strategy is then estimated as given in Eq. (4). Where $\:{CRm}_{k}^{g-1}$ is the $\:{CRm}_{k}$ value of previous generation.

4 $$\:{CRm}_{k}^{g}={CRm}_{k}^{g-1}+{\varDelta\:CRm}_{k}^{g}$$

**Fig. 3:**
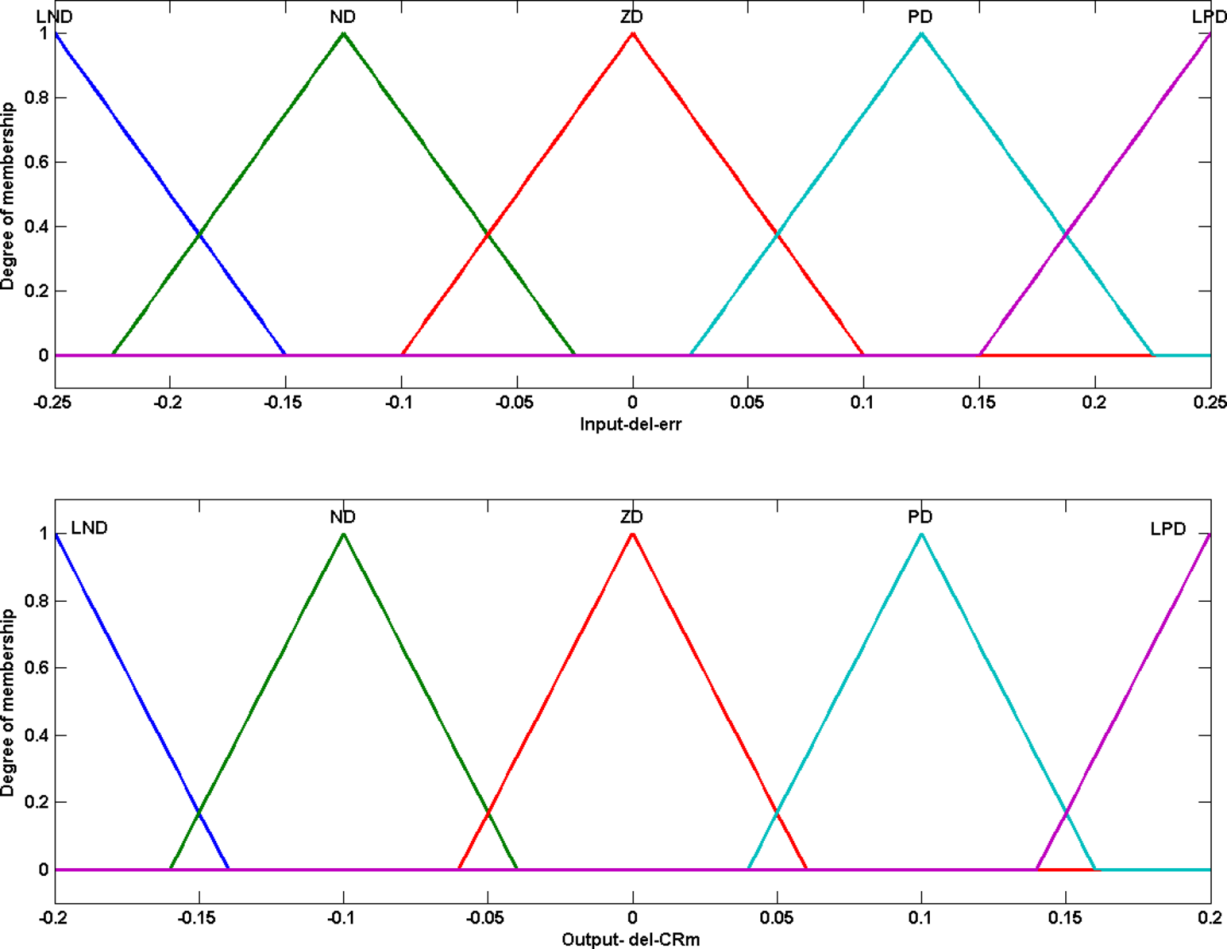
Fuzzy variables for $\:CRm$ adaptation. (a) Input variable ($\:{\varDelta\:err}_{g}$); (b) Output variable $\:{(\varDelta\:CRm}_{k}^{g}$)

Figure 3 gives the range and the degree of membership for fuzzy variables and is defined through triangular function.

#### Scaling factor adaptation using FS

The fuzzy system designed to adapt the scaling factor value is described in this section. The third control parameter in DE algorithm is the scaling factor $\:F$ value, which controls the difference vector amplification. A larger value of $\:F$ is necessary at initial stages where a diversified search is needed. At later stages when the search process sharpens smaller value of $\:F$ is needed.

In F-MAD algorithm $\:F$ value is generated initially through normal distribution $\:N\left(\text{0.5,0.3}\right)$. The scaling factor values are generated and used in each target vector in the current population. Consequently, from second iteration FS is used to adapt the mean of scaling factor $\:Fm\:$automatically based on the location of individuals and population diversity.

Mamdani’s fuzzy system with two input and one output is used for adapting scaling factor $\:F$. The two inputs to FS are diversity error ($\:{\varDelta\:err}_{g}$) at $\:{g}^{th}$ generation, calculated from (3) and the second input is the maximum of absolute difference in objective function values between the population best individual in the current generation and individual i, difference calculated across all the objectives as:

5 $$\:\varDelta\:{f}_{g}^{i}=max\left|{f}_{g}^{n}\left({x}_{best}\right)-{f}_{g}^{n}(\left.{x}_{i})\right|\right.$$

where $\:n=\text{1,2},\dots\:.k.$ the number of objectives.

Adapting $\:F$ with respect to the objective with maximum difference in fitness value aids in fine tuning the search and to converge to pareto set (True Pareto front). The best individual in the current generation $\:{f}_{g}\left({x}_{best}\right)$ is identified using the fast non-dominated sort approach^39^ where, the fitness value of the target vector is calculated and the individuals are divided into fronts using fast non-dominated sorting technique and one solution is selected randomly from the first non-dominated front as best individual in the current generation.

$\:{\varDelta\:err}_{g}$ and $\:\varDelta\:{f}_{g}^{i}$ are given as fuzzy inputs. The output of the Fuzzy system is the change in mean for $\:F$ value represented as $\:\:\varDelta\:F{m}_{g}^{i}$, with respect to each individual in the population. Thus, the mean value of normal distribution for scaling factor $\:F$ for $\:{i}^{th}$ individual in current generation is estimated by:

6 $$\:F{m}_{g}^{i}=F{m}_{g-1}^{i}+\varDelta\:F{m}_{g}^{i}$$

Through above adaptation, optimal $\:F$ values are generated to control the amplification as desired to reach True front. The $\:F$ value is bounded in the range [0.4,1] and $\:F$ value is varied for each solution using its location from the best individual in the current population and based on population diversity.

$\:{\varDelta\:err}_{g}$ input is described by linguistic variables as in FS for $\:CR$ adaptation. The objective fitness difference input $\:\varDelta\:{f}_{g}^{i}$ is divided in to linguistic variables the Zero Difference (Z), the Positive Difference (PD), and Largely Positive Difference (LPD) bounded by range [0,1] since it is an absolute value.

The Fuzzy System output, change in scaling factor mean $\:\varDelta\:Fm$ is defined by linguistic variables the Largely Negative Difference (LND), the Negative Difference (ND), the Zero Difference (Z), the Positive Difference (PD), and the Largely Positive Difference (LPD).

**Fig. 4:**
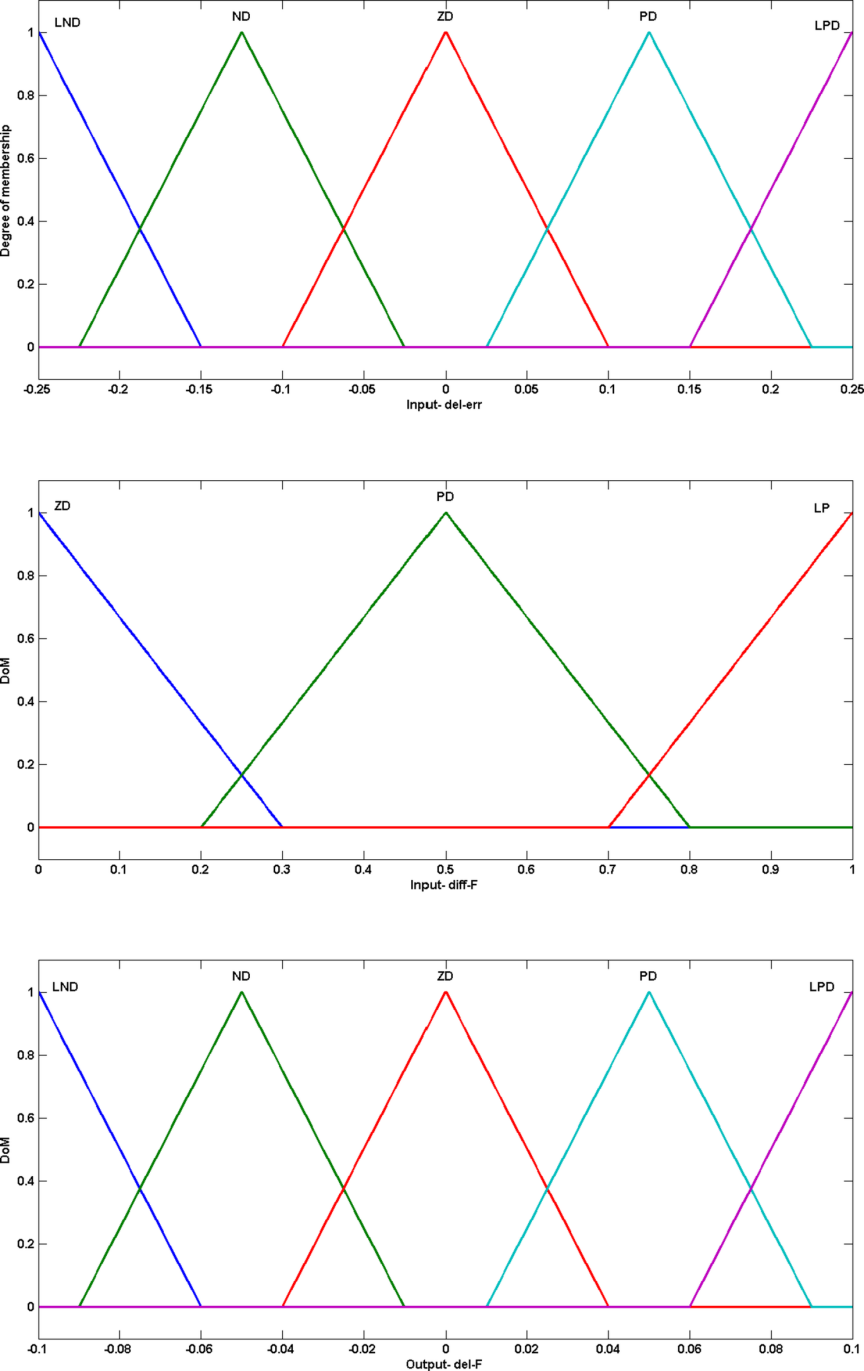
Fuzzy variables for $\:Fm$ adaptation. (**a**) Input variable 1 ($\:{\varDelta\:err}_{g}$); (**b**) Input variable 2 ($\:\varDelta\:{f}_{g}^{i}$); (**c**) Output variable $\:(\varDelta\:Fm)$

The implication rule set for scaling factor FS is given in Table 2. The rule set is developed such that when input $\:{\varDelta\:err}_{g}$ is PD and input $\:\varDelta\:{f}_{g}^{i}$ is LPD, it indicates that reference diversity ($\:ref$) is higher than current population diversity and $\:{i}^{th}$ individual is far away from the best individual. Thus, in order to improve exploration and to enhance diversity the output $\:F{m}_{g}^{i}$, mean of normal distribution for scaling factor to be increased with output value PD. Figure 4 gives the range and the degree of membership for the fuzzy variables and is defined through triangular function.

**Table 2:** Fuzzy rules for adapting scaling factor.

Δ*f*	Δerr				
	LND	ND	ZD	PD	LPD
LPD	LND	ND	ZD	PD	LPD
PD	LPD	ND	ZD	ND	LND
ZD	LND	ND	LND	ND	ZD

Thus, through the above method of FS based $\:F$ adaptation, larger $\:F$ value produced for individual away from population best solution to search wider region and smaller $\:F$ values produced for individuals located near to best individual in order to fine tune search to attain true front. The above method to self-adapt the scaling factor $\:F$ in addition to $\:CR$ adaptation enhances the exploration and exploitation nature of each solution in the population.

### Selection

The procedure followed to select fitter solutions to proceed with evolution is detailed in this section. Using the above trial vector and control parameter self-adaptation methods, trial vector is generated and then target vector and trial vectors are combined to 2NP solutions. Selection of NP potential solutions for next generation performed using non-domination based method improved with controlled elitism and dynamic crowding distance techniques. First, non-dominated sorting over combined population of 2NP solutions is performed where, solutions are segregated into separate fronts based on their non-domination factor. NP solutions are selected from these fronts using controlled elitism method^1^. Controlled elitism technique improves the lateral diversity in objective space with respect to Pareto Front, which is affected due to elitism based selection in fast non-dominated sort and due to local search procedure. It uses a distribution to derive the maximum number of individuals to be selected from each non-dominated front as:

7 $$\:{N}_{i}=N\frac{1-r}{1-{r}^{k}}{r}^{i-1}$$

$\:{N}_{i}$ is the output which is number of solutions to be selected from $\:{i}^{th}$ front ($\:i=\text{1,2},.k$) for the next generation of size $\:NP$. Reduction rate $\:r$(< 1) is specified by the user. Since the rate r is less than one, more individuals are selected from first front and from remaining fronts number of solutions selected is reduced and this solutions from all fronts are allowed to participate thus controlling elitism and improving diversity in objective space.

After deriving required number of solutions from each front, to remove excess individuals from the front “Dynamic Crowding Distance (DCD)” strategy^40^ is used to maintain uniform diversity. For all individuals in the front DCD value is calculated. An individual with lowest DCD value is removed from population. DCD value is recalculated for the remaining individuals and one with lowest value is removed. This process continued till we obtain $\:{N}_{i}$ solutions from the front. DCD is calculated as:

8 $$\:{DCD}_{i}=\frac{{CD}_{i}}{{log}\left(\frac{1}{{Vcd}_{i}}\right)}$$

Where $\:{CD}_{i}$ is the crowding distance of $\:{i}^{th}$ individual and is calculated as indicated in NSGA-II algorithm:

9 $$\:{CD}_{i}=\frac{1}{{N}_{obj}}\sum\:_{k=1}^{{N}_{obj}}|{f}_{i+1}^{k}-{f}_{i-1}^{k}|$$

Where, $\:{N}_{obj}$ variable denotes the number of objectives. $\:{f}_{i+1}^{k}$ and $\:{f}_{i-1}^{k}$ are the $\:{k}^{th}$ objective values for neighbours of $\:{i}^{th}$ individual. $\:{Vcd}_{i}$ variable in (8) is the variance among crowding distance values with respect to neighbours of $\:{i}^{th}$ individual and is estimated using:

10 $$\:{Vcd}_{i}=\frac{1}{{N}_{obj}}\sum\:_{k=1}^{{N}_{obj}}\left(\right|{f}_{i+1}^{k}{{-f}_{i-1}^{k}|-{CD}_{i})}^{2}$$

The above controlled elitism and dynamic crowding distance based non-dominated sorting selection, improves the convergence and diversity properties of the algorithm. Thus, control parameter adaptation using population diversity and improved selection measure balances both decision and objective space search properties and make the algorithm robust.

### Controlled local search

In this section, the proposed controlled local search to improve the exploitation characteristics is detailed in this section. Adaptation of $\:CRm$ and $\:Fm\:$based on diversity control improves the exploration characteristics of search algorithm. The diversity control prevents the convergence properties of the population. To aid in convergence, during the later stages of search process population diversity has to be reduced through the exploitation of promising solutions in the population. The Local search based algorithms is a renowned method for exploitation. The local search procedure searches for a better solution within the boundaries of a chosen solution and this locally improved solution will be added to the population set to compete with other solutions in the upcoming generations. This procedure improves convergence properties through exploitation.

In the proposed F-MAD algorithm, the local search procedure is called once in every predefined $\:{\:L}_{cnt}$generation with a pre specified number of function evaluations $\:Lfeval$ at the proximity of best individual. The best individual is chosen randomly among the individuals which is selected from first front (best non-dominated front) while performing fast non-dominated sorting. When an individual that dominates this best is identified or is found to dominate any of the solutions selected from the first front, a random individual is then replaced with a locally better solution found through local search and non-dominated sort procedure is repeated and fronts are updated.

Local search with elitism at the current population best individual has a higher chance of escaping from getting stuck to local optimum and will guide the population to attain true pareto-optimal front, thereby improving the exploitation. Controlled local search methodology of once in every $\:{\:L}_{cnt}$generation is performed to avoid too much of exploitation which may also lead to the state of premature convergence and to better avoid computational overhead. The pseudo code for local search is given below.

**Pseudocode 1:**
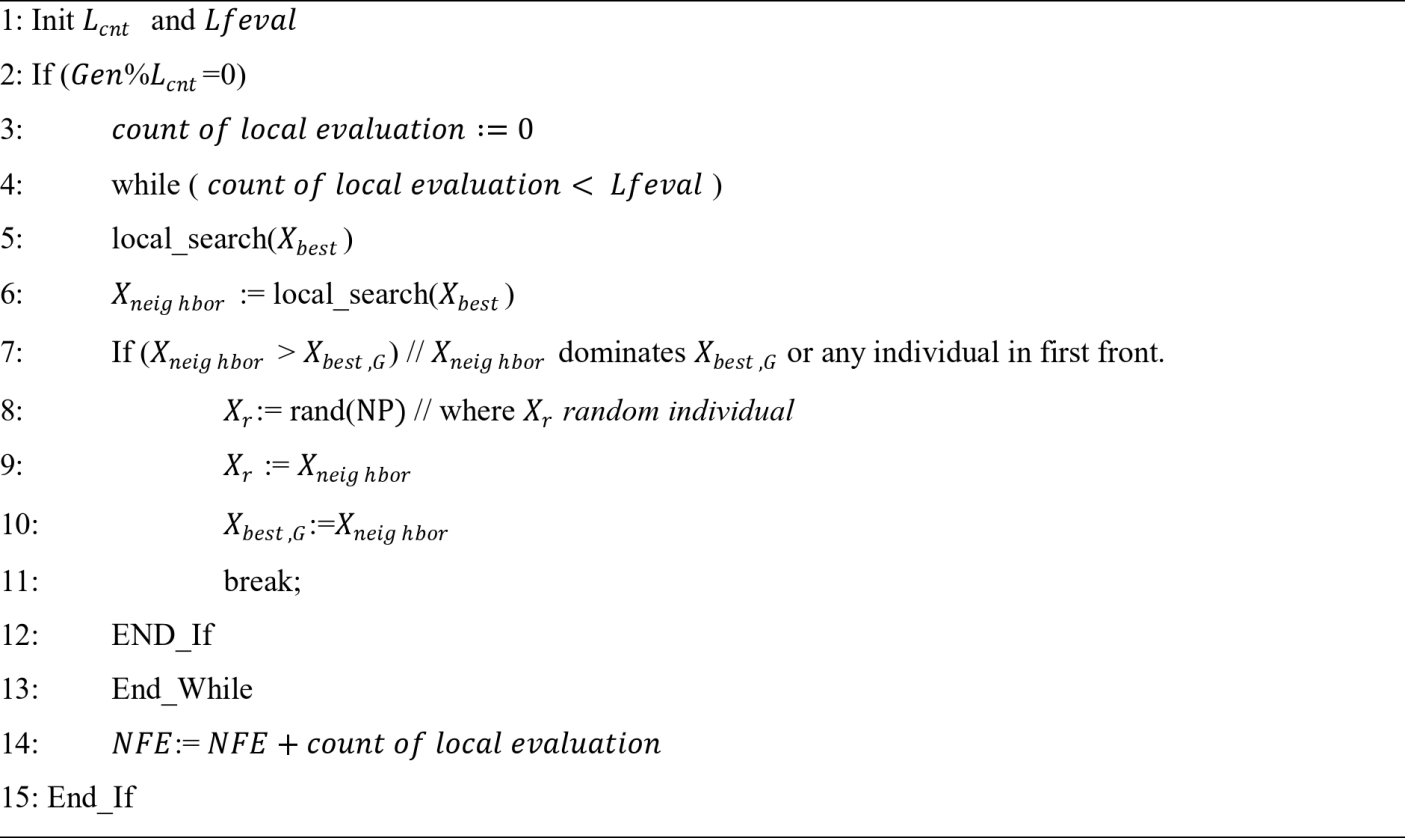
Controlled local search.

## Experimentation section- results and discussion

The performance of the proposed method F-MAD is evaluated as below. The initial parameter values of algorithm are listed in Sect. 3.1. The dataset details are given in Sect. 3.2. The metrics used for performance evaluation is given in Sect. 3.3. The results are detailed in subsequent Sect. 3.4 and 3.5.

The experiment is carried out on Matlab (R2019a) using a laptop with intel core i7 processor, 16 GB RAM. The algorithm is initialized to values listed in Table 3 and executed for specific function evaluations and runs. IGD metric is used as quality indicator and the IGD value is calculated for the obtained solutions. The best, worst and mean IGD values over the runs are listed for comparison studies with other SOTA algorithms. To further proves the effectiveness of the proposed algorithm statistical analysis is performed and the results are provided.

### Initial setup for Experimentation

Population size $\:NP$ for CEC 2009 test suite and for the DTLZ test problems are set as 50 and 300 respectively. $\:CRm\:$and $\:Fm$ are initialized to 0.5. Reference diversity $\:refdiv=0.15$, Learning Period $\:(LPe$) is set as 50. Number of Function Evaluations (NFE) is set as 300,000 and 90,000 for CEC 2009 and DTLZ test problems respectively and each problem is executed up to 30 and 20 runs for CEC and DTLZ problems. The local search count $\:{L}_{cnt}$=30 and local search evaluation is set as $\:Lfeval=$300. The above setup is consistent with other comparison algorithms. The parameter values are listed in Table 3.

**Table 3:** Initial parameter setup for F-MAD algorithm.

Parameters	F-MAD	
	Values Initialized for CEC Test Problems	Values Initialized for DTLZ Test Problems
Population size ($\:NP)$	50	300
Initial Crossover rate mean ($\:CRm)$ value	0.5	0.5
Standard deviation value-crossover rate generation	0.1	0.1
Initial Scaling factor mean ($\:Fm)$ value	0.5	0.5
Standard deviation value-scaling factor generation	0.3	0.3
Reference diversity ($\:refdiv)$	0.15	0.15
Learning Period ($\:LPe)$	50	50
Reduction rate ($\:r)$	0.55	0.55
Number of Function Evaluations ($\:NFE)$	300,000	90,000
Number of trials/runs	30	20
$\:{LS}_{cnt}$	30	30
$\:{LS}_{feval}$	300	300

### Test suite

To evaluate the performance of the proposed memetic algorithm F-MAD, 17 benchmark test problems considered such as CEC 2009 test problems^41^ and DTLZ test problems^42^. From CEC 2009 test suite first ten (UF1 to UF10) test problems are considered for experimentation where all are unconstrained test problems and from DTLZ test suite DTLZ1 to DTLZ7 test problems are taken and all DTLZ test problems are three objective problems.

### Metrics used for performance evaluation

To analyze the efficiency of the proposed F-MAD algorithm in solving the above challenging MooPs, quality indicator Inverted Generational Distance (IGD) and statistical non-parametric procedure, Friedman rank test are used.

IGD^43^ is used to calculate distance between the obtained front and True Front. Lower the IGD value implies how close obtained front to the True Front. To calculate IGD, two variables A and B are taken, where ‘A’ is the uniformly spread solution set across the true front and ‘B’ is approximation to the obtained front. IGD is estimated as:

11 $$\:IGD\left(A,B\right)=\frac{{\sum\:}_{x\in\:A}d(x,B)}{\left|A\right|}$$

Where $\:d(x,B)$ is the minimum Euclidean distance.

Statistical evaluation is also performed to analyze capability of the algorithm using nonparametric test - Friedman rank test^44^. Friedman test is a multiple sign test for multiple comparisons. The above test is implemented using IBM SPSS tool and $\:p$-value is computed. $\:\alpha\:$ the significance level is assigned as 0.05 and 0.1 for CEC and DTLZ problems respectively. If the $\:p$-value is less than $\:\alpha\:$ then, it implies that significant difference exists among the algorithms taken for comparison and null hypothesis is rejected thus stating proposed algorithm is better. If $\:p$-value is higher than $\:\alpha\:$ then it implies that between algorithms taken for evaluation there is not much significant variation.

### CEC 2009 test problems – results and its discussion

The statistics best, worst, mean and standard deviation values of IGD metric for CEC 2009 Test Problems using proposed algorithm is listed in Table 4, which is taken among 30 independent trials. It can be observed that the solutions generated using F-MAD algorithm is clustered and with less variance, proving the consistency of the method. Which can be better interpreted in the visual plots given below.

The mean IGD values derived by 30 independent runs for CEC problems is listed in Table 5 and a comparative analysis of IGD metric results is performed with our earlier method Fuzzy Adaptive Multi-objective Differential Evolution with Diversity Control (FAMDE-DC)^33^ and 17 other algorithms whose IGD values are derived from^45^. The results are further compared with recent algorithms Differential Evolution based Noise handling Optimization algorithm (NDE)^36^ and Multi-objective Memetic Differential Evolution algorithm (MOMDE)^46^ the details of all the algorithms taken for comparison is given in Table 5.

**Table 4:** Results - CEC 2009 test suite.

	UF1	UF2	UF3	UF4	UF5	UF6	UF7	UF8	UF9	UF10
Best	0.00250	0.00160	0.00290	0.00410	0.17800	0.01280	0.00140	0.000600	0.000400	0.00660
Worst	0.00430	0.00420	0.00480	0.00470	0.65300	0.03420	0.00420	0.00170	0.00340	0.00830
Mean	0.00350	0.00250	0.00360	0.00440	0.35700	0.02150	0.00300	0.00110	0.00120	0.00740
SD	0.000790	0.00110	0.000779	0.000270	0.24200	0.00790	0.00110	0.000403	0.00120	0.000723

**Table 5:** Algorithms taken for comparison studies.

Ref. no	Algorithm
^33^	Fuzzy Adaptive Multi-objective Differential Evolution with Diversity Control (FAMDE-DC)
^47^	Archive-based Micro Genetic Algorithm (AMGA)
^48^	Clustering Multi-objective Evolutionary Algorithm (Clustering MOEA)
^49^	Differential Evolution with Self-adaptation and Local Search for Constrained Multiobjective Optimization algorithm (DECMOSA-SQP)
^50^	Dynamical multiobjective evolutionary algorithm with decomposition technique (DMOEADD)
^51^	Generalized Differential Evolution 3 (GDE3)
^52^	Multiobjective evolutionary algorithm based on determined weight and sub-regional search (LiuLi Algorithm)
^53^	Multiobjective ABC (MOABC)
^54^	Multi-objective biogeography based optimization with ACO (MO-BBO_ACO)
^55^	MultiObjective Evolutionary Algorithm based on Decomposition (MOEA/D)
^56^	MOEA/D with Guided Mutation and Priority Update (MOEADGM)
^57^	Multi-objective evolutionary programming (MOEP)
^58^	Multi-objective teaching learning based optimization (MO-TLBO)
^59^	Multiple trajectory search (MTS)
^60^	Nondominated genetic algorithm with local search (NSGAIILS)
^45^	Multi-objectivemoth flame optimization (NS-MFO)
^61^	Orthogonal Multi-objective Evolutionary Algorithm (OMOEA-II)
^62^	Multiobjective Self-adaptive Differential Evolution algorithm with objective-wise learning strategies (OW-MOSaDE)
^36^	Differential Evolution based Noise handling Optimization algorithm (NDE)
^46^	Multi-objective Memetic Differential Evolution algorithm (MOMDE)

Table 6 lists the results of comparative studies. From the results shown in Table 6, it is evident that F-MAD is able to solve all the UF test instances effectively than other methods except problem UF5 and UF6 and the results are better than the FAMDE-DC method where only single FS is used without a local search procedure. The adaptation of the control parameter values based on the population diversity and the position of the solutions in the current population when compared to best individual makes the exploration in a more efficient way. Moreover, the proposed controlled local search technique improves the exploitation capabilities of the F-MAD algorithm.

**Table 6:** Mean IGD values - CEC 2009 problems.

Algorithm	UF1	UF2	UF3	UF4	UF5	UF6	UF7	UF8	UF9	UF10
F-MAD	**0.00350**	**0.00250**	**0.00360**	**0.00440**	0.35700	0.02150	**0.00300**	**0.00110**	**0.00120**	**0.00740**
FAMDE-DC	0.00495	0.00263	0.00397	0.00480	1.4400	0.02790	0.00730	0.00179	0.00149	0.00896
AMGA	0.03590	0.01620	0.07000	0.04060	0.09410	0.12900	0.05710	0.17100	0.18900	0.32400
Clustering MOEA	0.02990	0.02280	0.05490	0.05850	0.24700	0.08710	0.02230	0.23800	0.29300	0.41100
DECMOSA-SQP	0.07700	0.02830	0.09350	0.03390	0.16700	0.12600	0.02420	0.21600	0.14100	0.37000
DMOEADD	0.01040	0.00679	0.03340	0.04270	0.31500	0.06670	0.01030	0.06840	0.04900	0.32200
GDE3	0.00534	0.01200	0.10600	0.02650	0.03930	0.25100	0.02520	0.24900	0.08250	0.43300
LiuLi	0.00785	0.01230	0.01500	0.04350	0.16200	0.17600	0.00730	0.08240	0.09390	0.44700
MOABC	0.00618	0.00484	0.05120	0.05800	0.07780	0.06540	0.05570	0.06730	0.06150	0.19500
MO-BBO_ACO	0.00579	0.00784	0.06930	0.03590	0.03790	0.05530	0.02140	0.09930	0.11700	0.17900
MOEA/D	0.00435	0.00679	0.00742	0.06390	0.18100	**0.00587**	0.00440	0.05840	0.07900	0.47400
MOEADGM	0.00620	0.00640	0.04290	0.04760	1.79000	0.55600	0.00760	0.24500	0.18800	0.56500
MOEP	0.05960	0.01890	0.09900	0.04270	0.22500	0.10300	0.01970	0.42300	0.34200	0.36200
MO-TLBO	0.00879	0.00784	0.06930	0.07010	0.09790	0.05530	0.06800	0.09930	0.16400	0.17900
MTS	0.00646	0.00615	0.05310	0.02360	**0.01490**	0.05920	0.04080	0.11300	0.11400	0.15300
NSGAIILS	0.01150	0.01240	0.10600	0.05840	0.56600	0.31000	0.02130	0.08630	0.07190	0.84500
NS-MFO	0.00421	0.00762	0.06720	0.03290	0.06290	0.04540	0.02020	0.06290	0.20000	0.43300
OMOEA-II	0.08560	0.03060	0.27100	0.04620	0.16900	0.07340	0.03350	0.19200	0.23200	0.62800
OW-MOSaDE	0.01220	0.00810	0.10300	0.05130	0.43000	0.19200	0.05850	0.09450	0.09830	0.74300
NDE	0.00443	0.00687	0.00749	0.06412	0.18107	0.00591	0.00452	0.05867	0.07912	0.47409
MOMDE	0.08554	0.03058	0.27123	0.04698	0.16901	0.07423	0.03367	0.19390	0.23400	0.62807

**Fig. 5:**
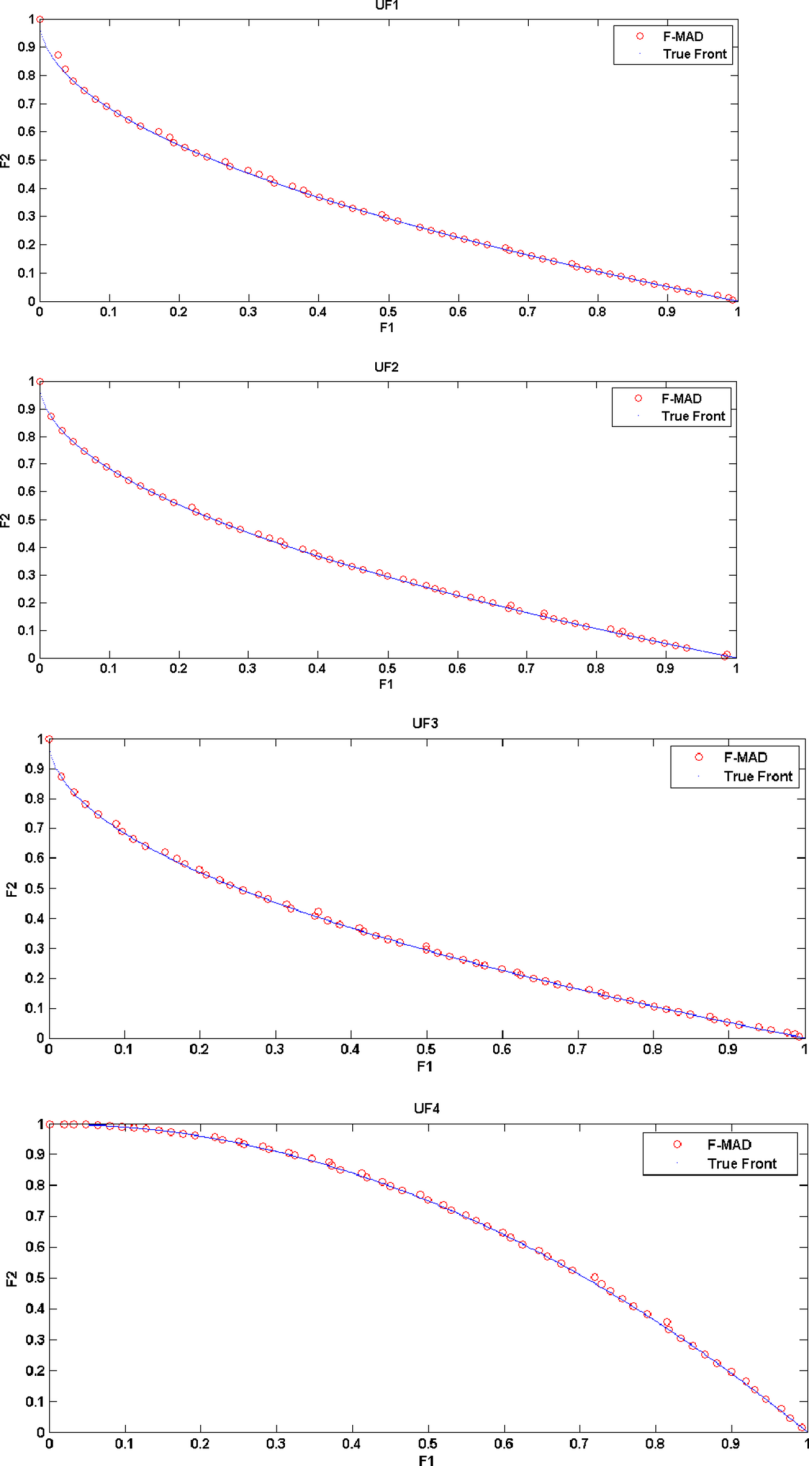

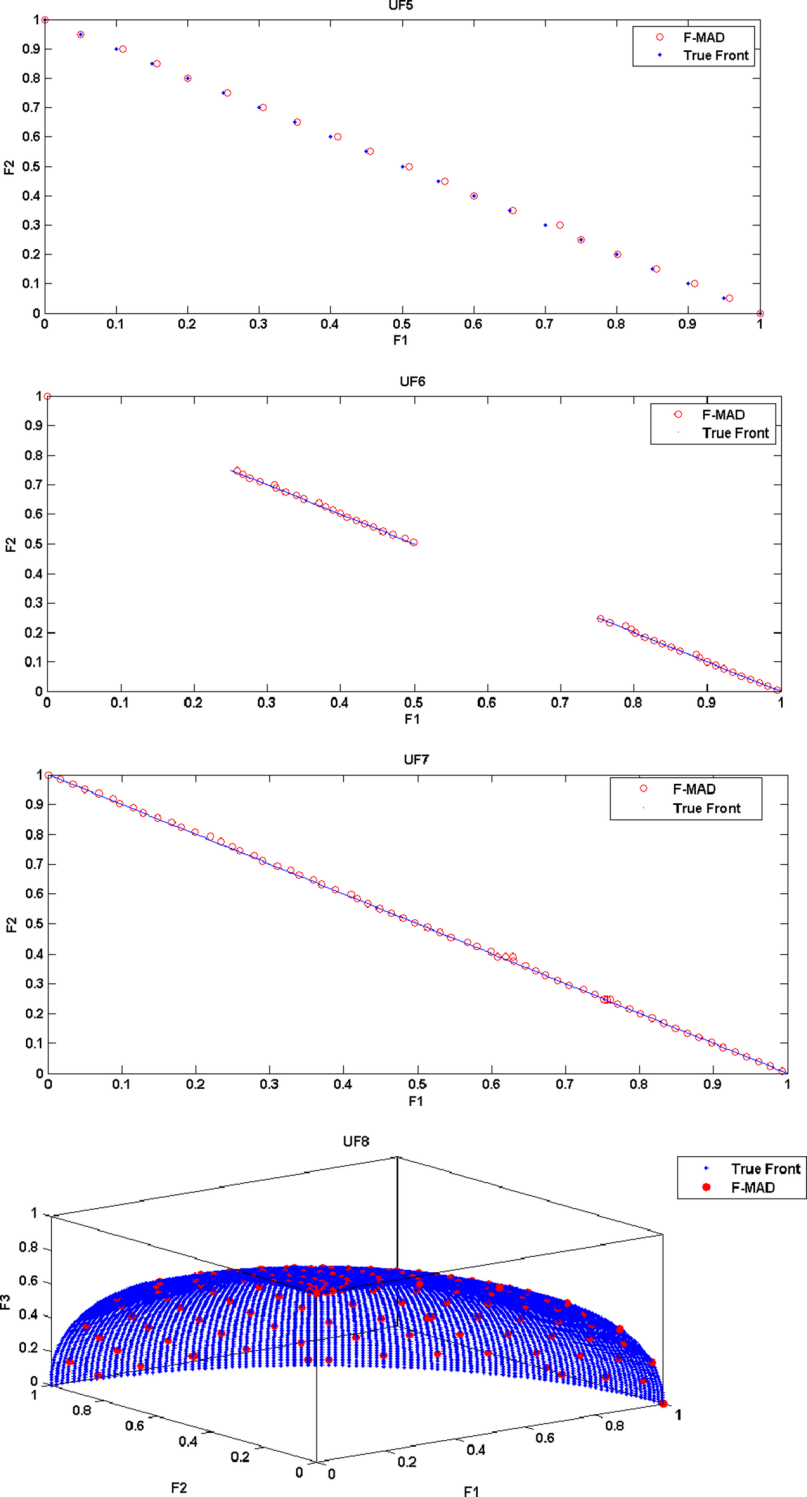

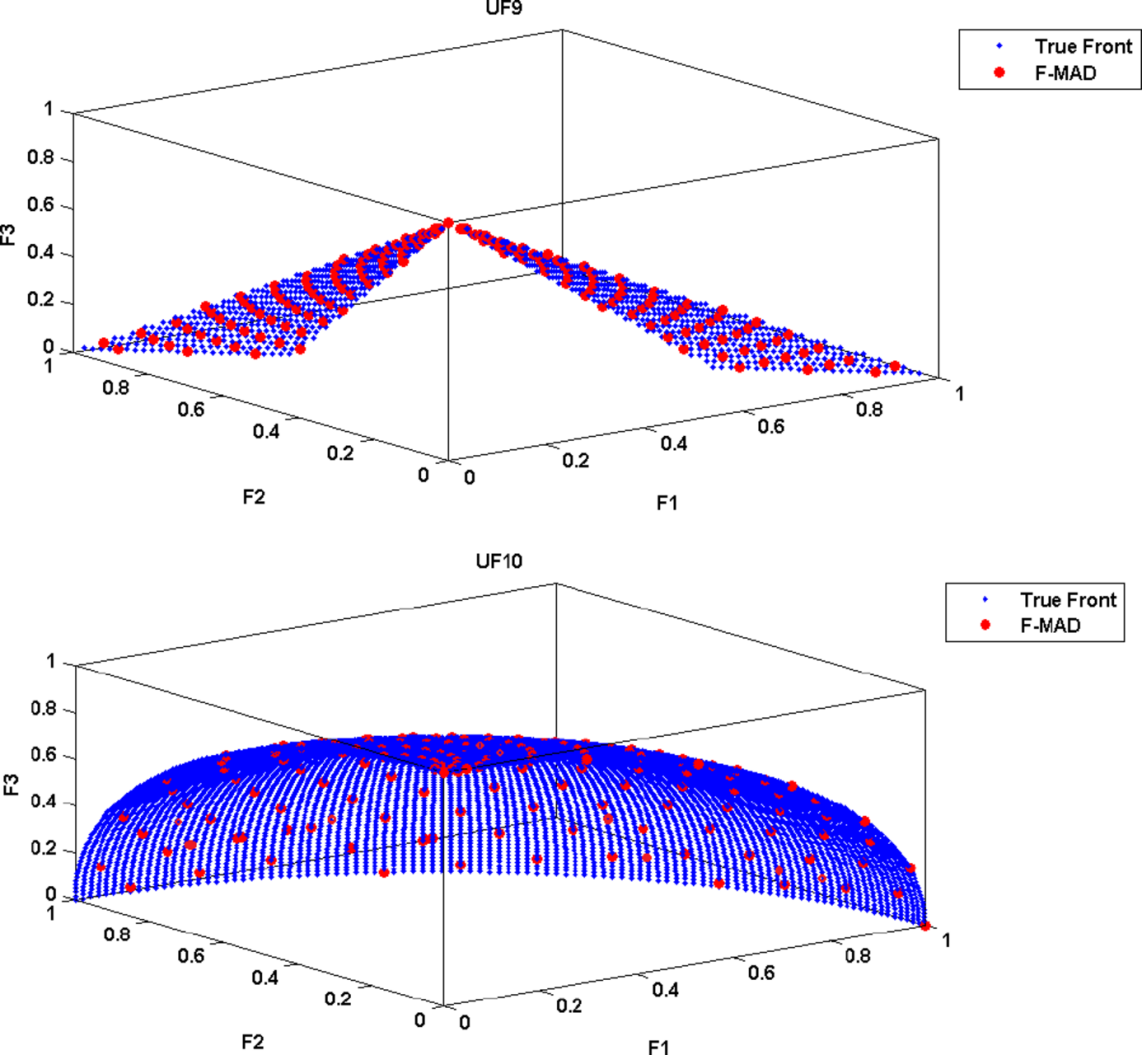
True and attained Pareto Fronts by F-MAD for CEC 2009 test suite UF1-UF10.

The True Front and attained Pareto Front using F-MAD algorithm for CEC 2009 test instances UF1 to UF10 is illustrated in Fig. 5, from which it is clear that the presented fuzzy based memetic method is able to solve test instances effectively and reach True Front with good IGD measure. The true front is a single convex curve for UF1-UF3 test problems and F-MAD algorithm converges well towards the pareto-optimal curve and the attained solutions are also spread uniformly. The optimal front for UF4 is a single concave curve and the F-MAD algorithm converges well towards the true front and the solutions are well spread along the front. Next, for UF5 test Problem the optimal front comprises $\:2N+1$ dispersed Pareto optimal solutions ($\:N=10$) and the attained front using F-MAD method is not approximated well towards optimal solutions and the mean IGD value is thus affected. For problem UF6, the optimal fronts are discontinuous comprising of an isolate point located at (0,1) and other two disconnected parts and the proposed algorithm identified the isolate point and the two other disconnected fronts with uniform distribution. For UF7 the optimal front is a straight line and F-MAD attains the optimal front with a better IGD measure. The other three problems namely, UF8, UF9 and UF10 are all three objective problems. The attained front using proposed F-MAD algorithm covers a larger part of the objective space with good approximation respect to the true Pareto optimal solutions.

(The experiment is carried out on MATLAB R2019a)

**Fig. 6:**
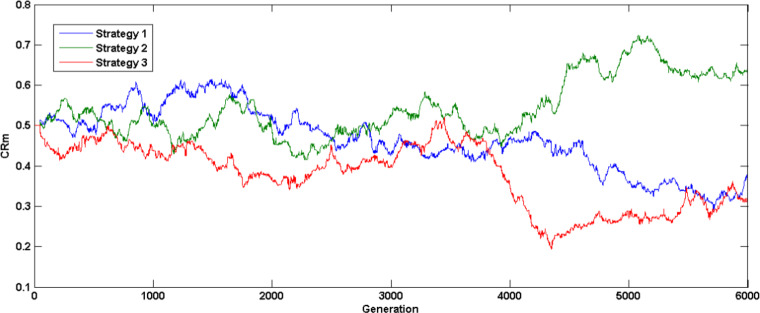
$\:CRm$ adaptation characteristics - UF4 Problem

**Fig. 7:**
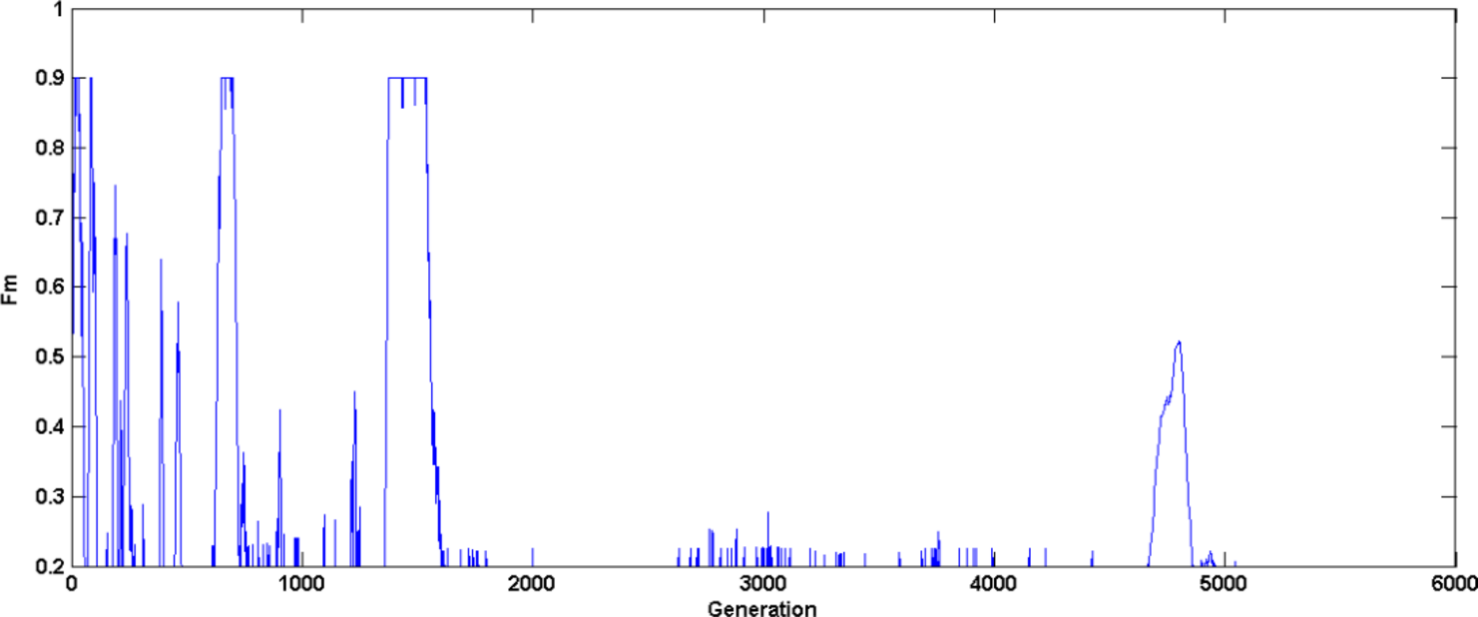
$\:Fm$ adaptation characteristics - UF4 Problem

**Fig. 8:**
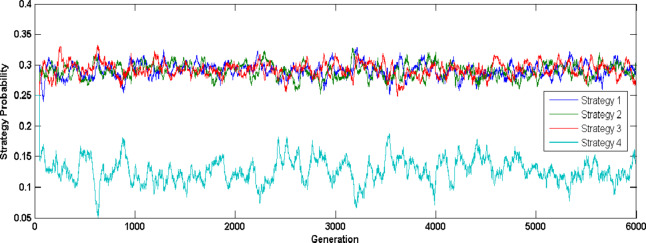
Strategy adaptation - UF4 Problem.

Figure 6 illustrates the crossover rate mean self-adaptation (UF4 test problem) for the first three strategies over various generations based on previous success index and according to population diversity. Figure 7 shows scaling factor mean adaptation where, initially higher values are generated to improve exploration later it’s reduced to fine tune the search towards optimal solution. Figure 8 illustrates the probability of various strategies (UF4 test problem) being used in various generations to generate trial vector based on their previous success index, it is evident that strategy four without crossover rate parameter doesn’t generate promising solutions thus compared to the other three strategies, the probability of fourth strategy being chosen for trial vector generation is less.

The Friedman rank statistical test results considering the mean IGD Values for CEC problems UF1 to UF10 are provided in Table 7, where the rank obtained by each algorithm against individual test problems is listed along with mean Friedman value and overall ranking of the algorithms. The proposed F-MAD performs better than other 20 algorithms and ranked first and $\:p$-value is 0.000, which is less than the significance level $\:\alpha\:$=0.05 thus show the efficiency of proposed memetic algorithm. The proposed F-MAD algorithm thus singnificantly outperforms the other multi-objective evolutionary algorithms taken for comparison. The proposed memetic algorithm exhibits this significance as the control parameters and trial vector generation startegies are rightly adapted based on the feedback given on the diversity and solution significance complemented with controlled local search.

**Table 7:** Friedman rank test results - CEC problems.

Algorithm	UF1	UF2	UF3	UF4	UF5	UF6	UF7	UF8	UF9	UF10	Friedman value	Rank
F-MAD	1	1	1	1	16	3	1	1	1	1	2.7	**1**
FAMDE-DC	4	2	2	2	19	4	3	2	2	2	4.2	2
MOEA/D	3	6	3	18	11	1	2	3	6	15	6.8	3
MTS	10	4	9	3	1	8	16	13	11	3	7.8	4
NS-MFO	2	9	11	5	4	5	9	5	17	12	7.9	5
MOABC	8	3	8	15	5	9	17	6	4	6	8.1	6
NDE	5	8	4	19	12	2	5	4	7	16	8.2	7
MO-BBO_ACO	7	10	12	7	2	6	11	11	12	4	8.2	7
DMOEADD	13	6	5	9	15	10	7	7	3	7	8.2	7
LiuLi	11	14	6	11	8	16	3	8	9	14	10	10
GDE3	6	13	18	4	3	18	14	19	8	12	11.5	11
MO-TLBO	12	10	12	20	7	6	20	11	14	4	11.6	12
MOEADGM	9	5	7	13	20	20	6	18	15	17	13	13
AMGA	17	16	14	8	6	15	18	14	16	8	13.2	14
DECMOSA-SQP	19	19	15	6	9	14	13	16	13	10	13.4	15
MOEP	18	17	16	9	13	13	8	20	20	9	14.3	16
NSGAIILS	14	15	18	16	18	19	10	9	5	20	14.4	17
Clustering MOEA	16	18	10	17	14	12	12	17	19	11	14.6	18
OW-MOSaDE	15	12	17	14	17	17	19	10	10	19	15	19
OMOEA-II	21	21	20	12	10	11	15	15	18	18	16.1	20
MOMDE	20	20	21	21	21	21	21	21	21	21	20.8	21

### Box-plot analysis

**Fig. 9:**
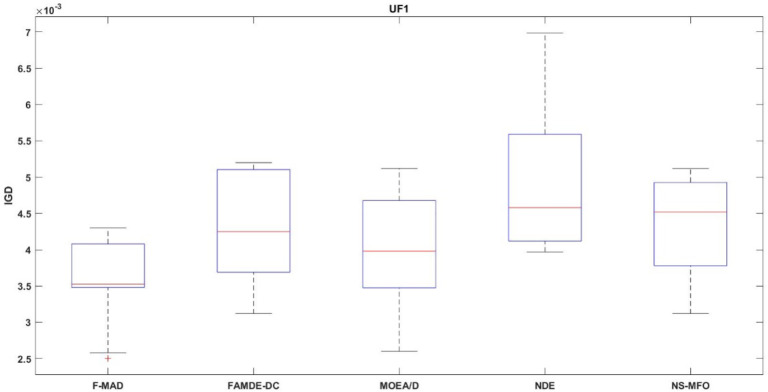
Box plot of UF1 test problem.

**Fig. 10:**
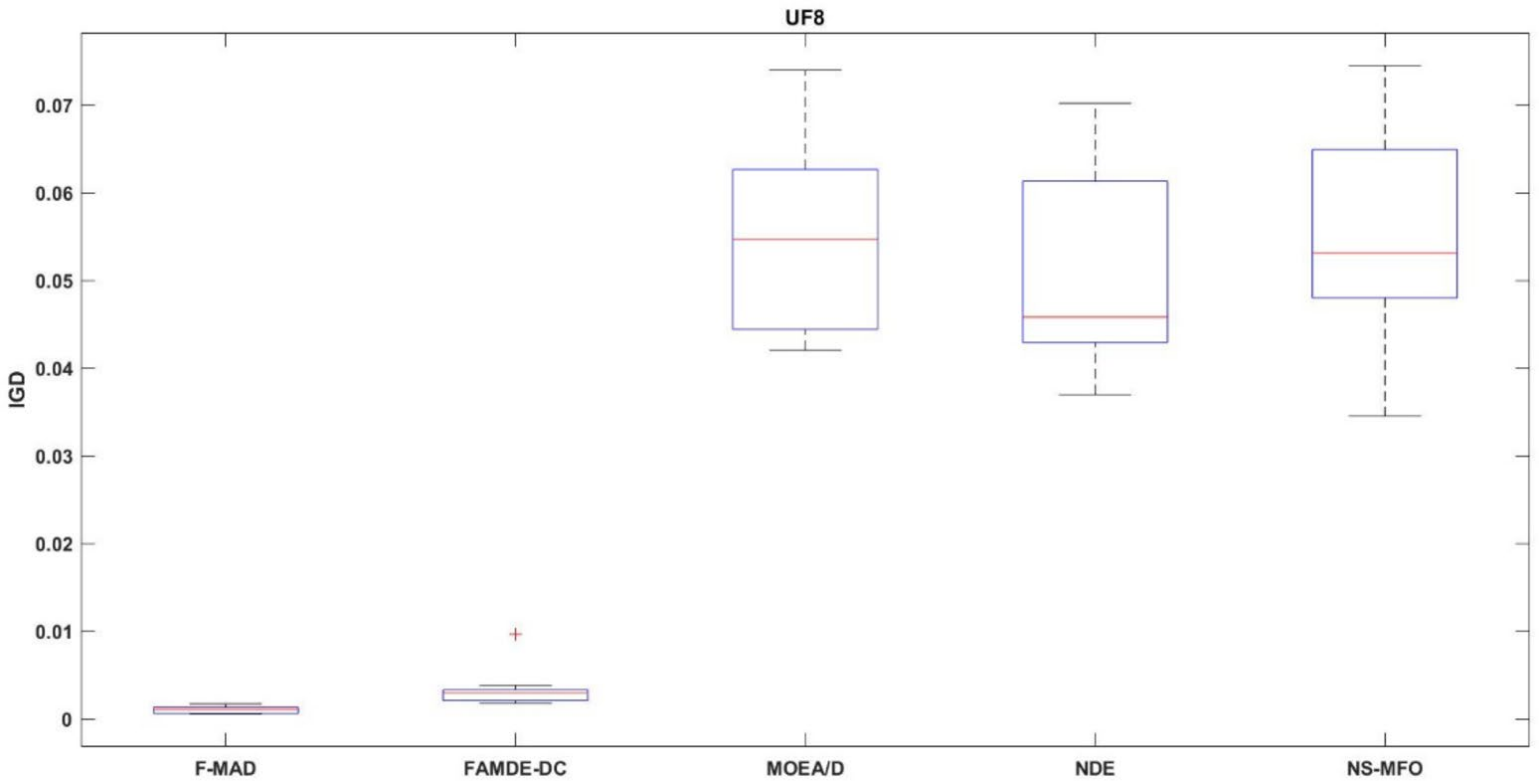
Box plot of UF8 test problem.

The IGD distribution of the algorithms with best performance for test problems UF1 and UF8 are shown in Figs. 9 and 10 respectively. Box-plot provides a visual representation to relate the central tend and how the fitness values are dispersed. Thus, the plot helps to better interpret about the performance of the algorithm. From the figures it is evident that F-MAD exhibits better performance, since the plot of the proposed F-MAD algorithm has minimum variability with consistent performance, which proves the robustness of the proposed algorithm.

### DTLZ test problems – results and its discussion

The statistics best IGD, worst IGD, mean IGD and standard deviation of IGD values for DTLZ Test Problems using proposed algorithm is listed in Table 8, which is taken across 20 independent trials. The attained solutions do have less variance and are consistent and clustered closely.

The mean IGD values derived by 20 independent runs for DTLZ problems is listed in Table 8 and the results are compared with FAMDE-DC algorithm and 5 other evolutionary algorithms and are listed in Table 9. The Mean IGD values of other methods are taken from MOEA/D-EAM^12^.

**Table 8:** Results **-** DTLZ test suite.

	DTLZ1	DTLZ2	DTLZ3	DTLZ 4	DTLZ 5	DTLZ 6	DTLZ 7
Best	0.00470	0.01360	0.01340	0.00050	0.00110	0.00080	0.01530
Worst	0.00740	0.01660	0.01650	0.00390	0.00150	0.00110	0.01840
Mean	0.00570	0.01530	0.01550	0.00240	0.00130	0.00094	0.01680
SD	0.00100	0.00110	0.00120	0.00130	0.000164	0.000151	0.00140

**Table 9:** Algorithms taken for comparison- DTLZ problems.

Ref. no.	Algorithm
^33^	Fuzzy Adaptive Multi-objective Differential Evolution with Diversity Control (FAMDE-DC)
^39^	Non-dominated sorting genetic algorithm NSGA-II
^36^	Differential Evolution based Noise handling Optimization algorithm (NDE)
^63^	MOEA/D with adaptive replacement strategy (MOEA/D-AGR)
^11^	Multi-objective evolutionary algorithm based on decomposition (MOEA/D)
^12^	MOEA/D with External archive matching strategy (MOEA/D-EAM)
^64^	MOEA/D based on differential evolution (MOEA/D-DE)
^46^	Multi-objective Memetic Differential Evolution algorithm (MOMDE)

From the results shown in Table 10, it is evident that F-MAD is able to solve all the DTLZ test instances effectively that other methods taken for comparison. The control parameter adaptation, strategy adaptation and balancing the exploitation through controlled local search techniques improves the robustness of the proposed algorithm. Thus, the attained results are better than other memetic or decomposition based variants of evolutionary algorithms taken for comparison.

**Table 10:** Mean IGD values - DTLZ problems.

Algorithm	DTLZ1	DTLZ2	DTLZ3	DTLZ 4	DTLZ 5	DTLZ 6	DTLZ 7
F-MAD	0.00570	0.01530	0.01550	0.00240	0.00129	0.00094	0.01680
FAMDE-DC	0.00630	0.01670	0.01630	0.00340	0.00130	0.00110	0.01720
NSGA-II	0.55600	0.05590	0.95000	0.06080	0.00167	0.00567	0.02451
NDE	0.07976	0.04995	0.08121	0.05489	0.00142	0.00118	0.01861
MOMDE	0.56712	0.06120	0.95122	0.09700	0.00176	0.00612	0.01987
MOEA/D-AGR	0.26700	0.05170	0.12500	0.05650	Unavailable		
MOEA/D	0.31100	0.05410	0.56400	0.08800	Unavailable		
MOEA/D-EAM	0.07970	0.04980	0.08070	0.05470	Unavailable		
MOEA/D-DE	0.21800	0.05280	0.23100	0.11500	Unavailable		

**Fig. 11:**
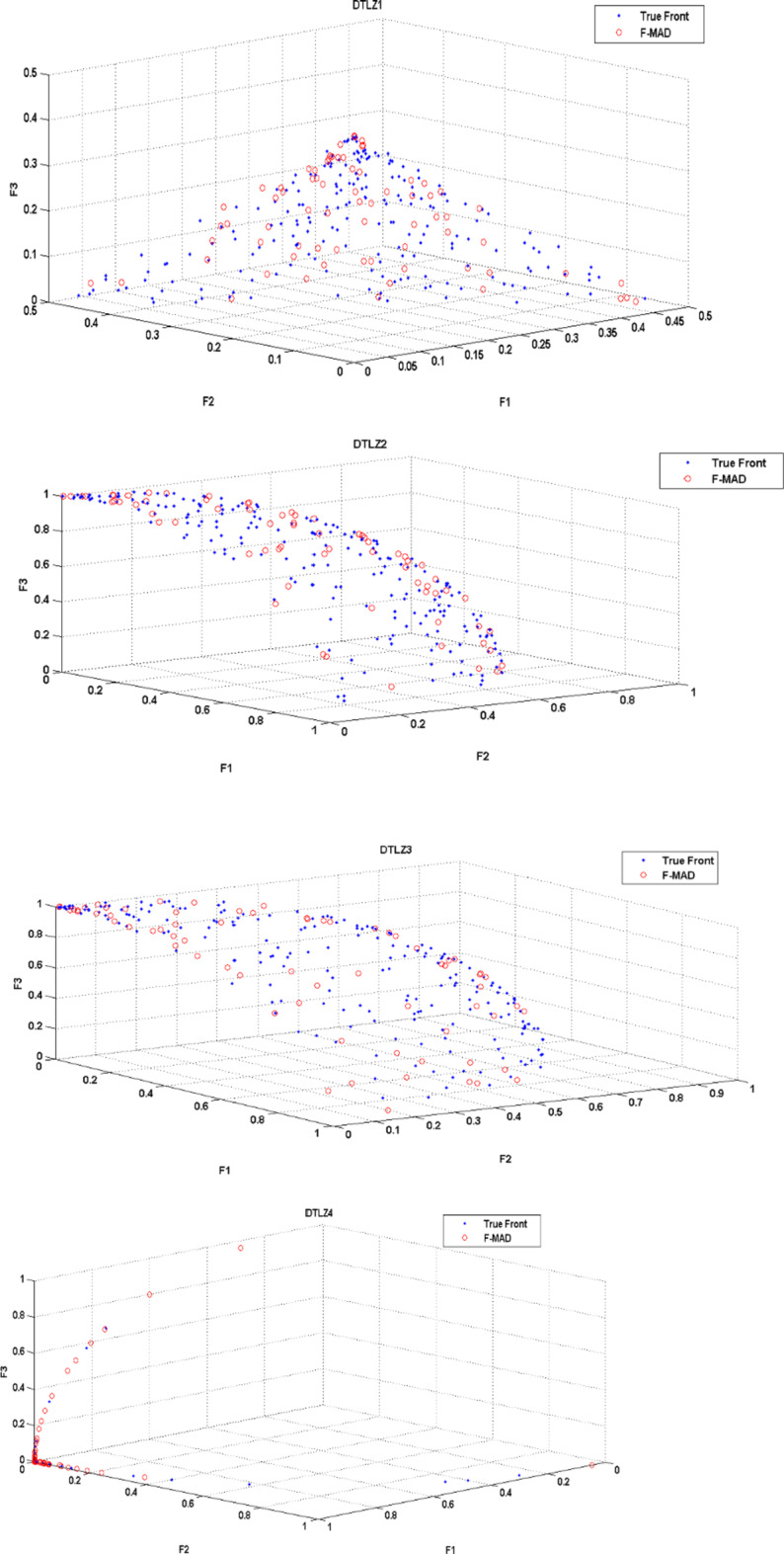

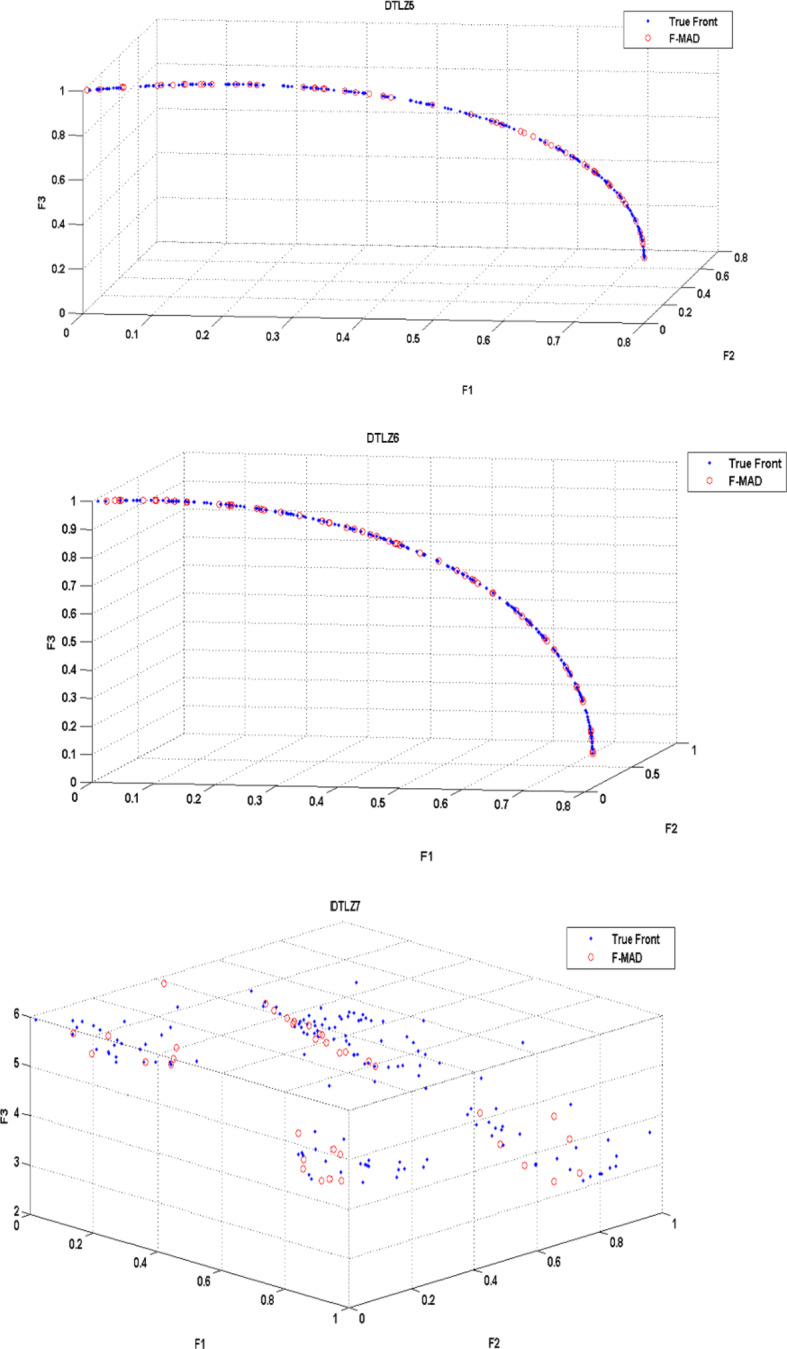
True and attained Pareto Fronts by F-MAD for DTLZ test suite DTLZ1-DTLZ7.

The True Front and attained Pareto Front using F-MAD algorithm for DTLZ1 to DTLZ 7 is illustrated in Fig. 11, from which it is evident that the proposed fuzzy based memetic algorithm is able to solve all DTLZ test instances effectively and approximates true front for all the DTLZ test problems. The pareto front obtained using the proposed F-MAD algorithm is well approximated with the true front for test problems DTLZ1, DTLZ2, DTLZ3. Next, for problem UF4 the larger number of solutions reached using F-MAD are covered in F-F2 and F3-F1 planes as in the case of True front. Problems DTLZ5 and DTLZ6 have optimal curve as True front and the attained front is a better approximation to it with uniform distribution. Lastly test problem DTLZ7 has 22 dimensions and true front includes four disconnected regions and the attained front includes solutions in all four regions with good distribution.

**Fig. 12:**
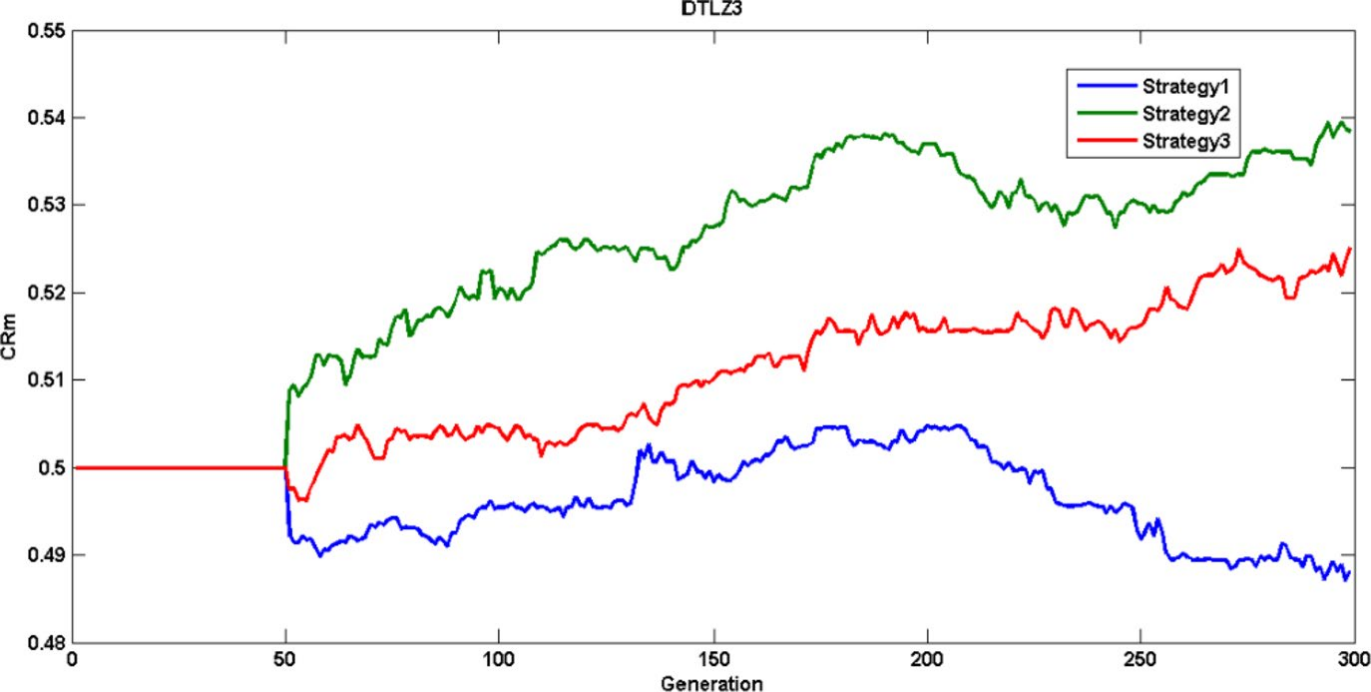
$\:CRm$ adaptation characteristics – DTLZ3 Problem

**Fig. 13:**
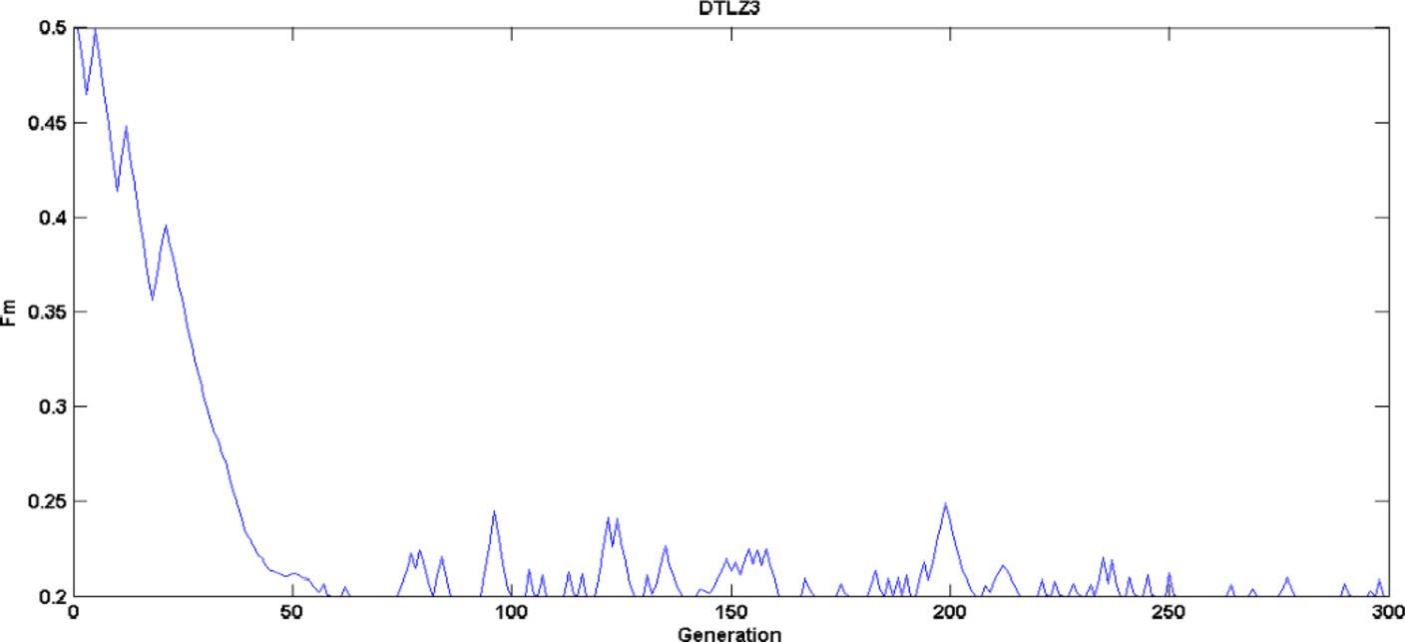
$\:Fm$ adaptation characteristics – DTLZ3 Problem

**Fig. 14:**
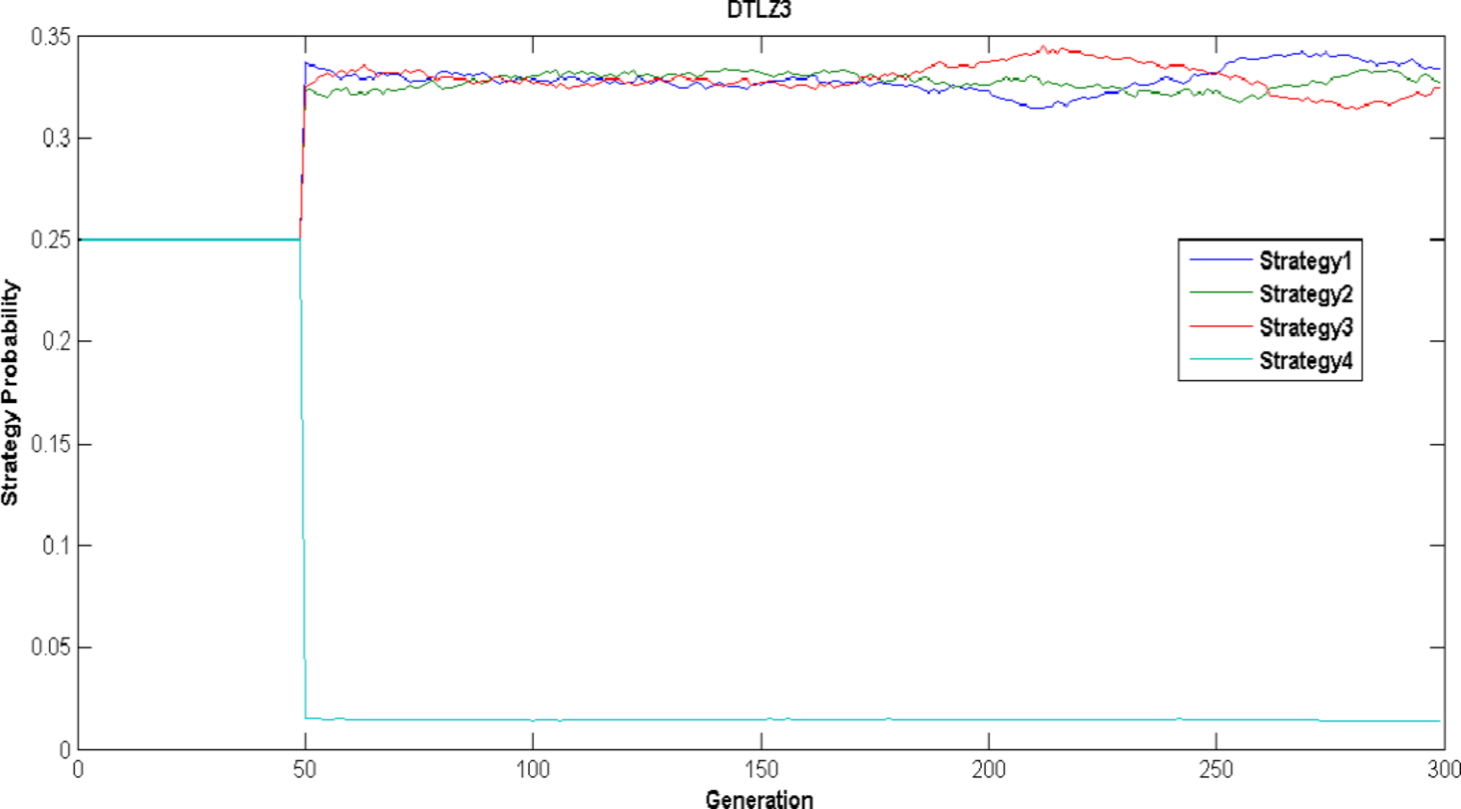
Strategy adaptation - DTLZ3 Problem.

Figure 12 shows the crossover rate mean self adaptation (DTLZ3 test problem) for the three strategies over various generations. Figure 13 illustrates the scaling factor mean adaptation for DTLZ3 problem and Fig. 14 represents the probability of various strategies (DTLZ3 test problem) being used in various generations to generate trial vector based on their previous success index, it is evident that strategy four without crossover rate and adaptation doesn’t generate potential individuals and probability of using it is less compared to other strategies.

### Box-Plot analysis

**Fig. 15:**
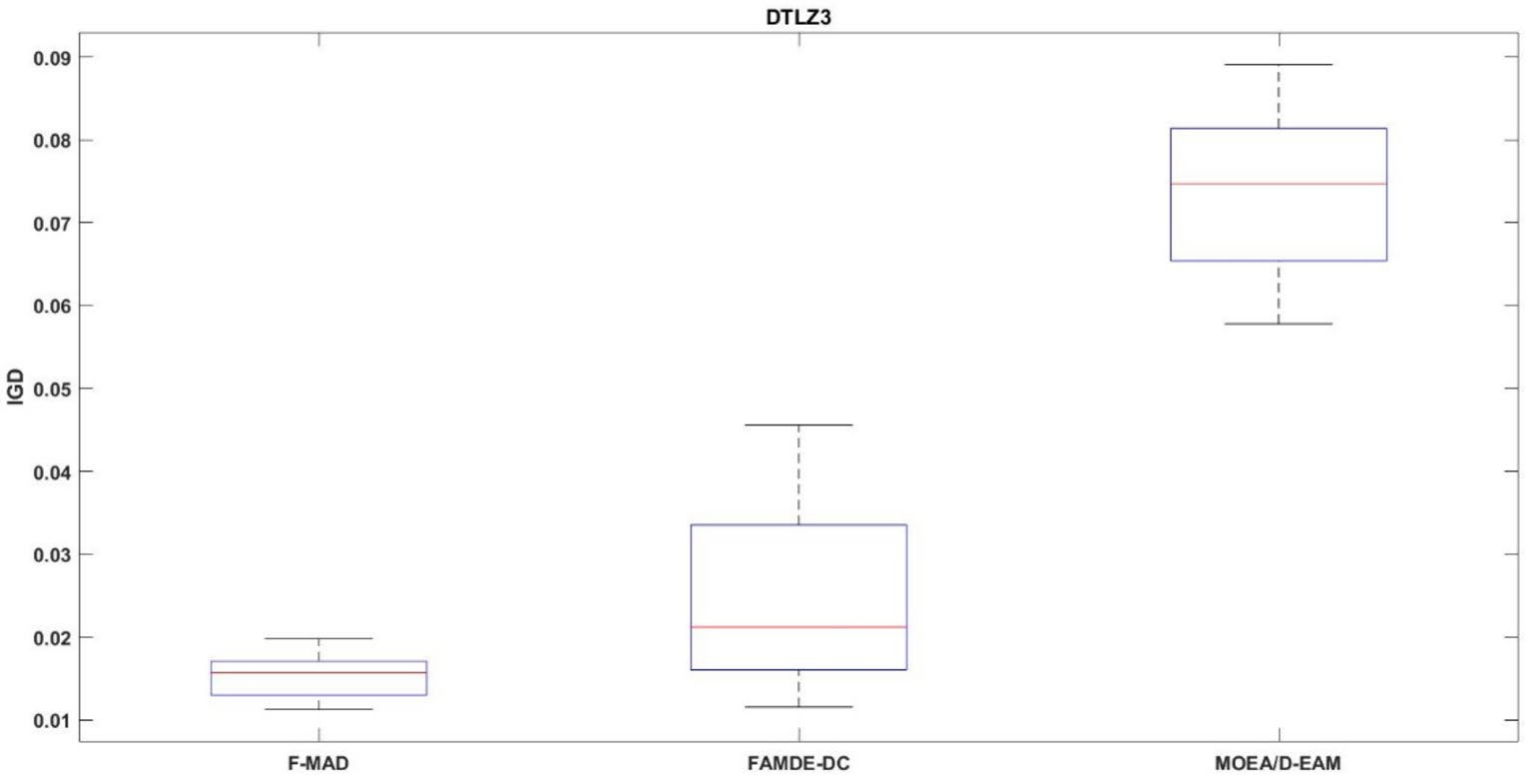
Box-plot DTLZ3 test problem.

The IGD distribution of the algorithms with best performance for test problem DTLZ3 is shown in Fig. 15. Box-plot provides a visual representation to relate the central tend and how the fitness values are dispersed. Thus, the plot helps to better interpret about the performance of the algorithm. From the figures it is evident that F-MAD exhibits better performance, since the plot of the proposed F-MAD algorithm has minimum variability with consistent performance, which proves the robustness of the proposed algorithm.

The Friedman rank statistical test results considering mean IGD Values for problems DTLZ1 to DTLZ4 are listed in Table 11. The proposed F-MAD performs better than other 8 algorithms taken for comparison and ranked first and $\:p$-value is 0.004, which proves the efficiency of the present algorithm statistically. These statistical test results further proves the effectiveness of the proposed adaptation and controlled local search based memetic algorithm.

**Table 11:** Friedman rank test results - DTLZ problems.

Algorithm	DTLZ1	DTLZ2	DTLZ3	DTLZ4	Friedman value	Rank
F-MAD	1	1	1	1	1	1
FAMDE-DC	2	2	2	2	2	2
MOEA/D-EAM	3	3	3	3	3	3
NDE	4	4	4	4	4	4
MOEA/D-AGR	6	5	5	5	5.25	5
MOEA/D-DE	5	6	6	8	6.25	6
MOEA/D	7	7	7	7	7	7
NSGA-II	8	8	8	6	7.5	8
MOMDE	9	9	9	9	9	9

## Case study – trajectory optimization

To further assess the performance of the proposed F-MAD algorithm, it is applied to solve a real-world multi-objective optimization problem. The introduction to test problem, its objective functions and results attained are discussed in this section. The real-world optimization problem chosen is related to the field of Mobile Edge Computing (MEC) system using Unmanned Aerial Vehicle (UAV)^65,66^, the trajectory optimization.

Minimizing energy and maximizing area covered are common optimization objectives related to this field. The multi-objective problem for optimization is minimizing energy consumption $\:\left(EC\right)$ and minimize process priority notifier $\:\left(PPN\right)$^65^. The problem formulation is given below.

12 $$\:minimize\{EC,PPN\}$$

Subject to:

Constraint 1: $$\:L=\left\{\begin{array}{c}1,\:if\:min\left(distance\right)\\\:0,\:\:\:\:\:\:\:\:\:\:\:\:\:\:otherwise\:\end{array}\right.$$

where, $\:L$ is the link connecting IoT device with stop position of UAV. The IoT devices connects through the link to the nearest stop position, given as $\:\text{m}\text{i}\text{n}\left(distance\right)$. Thus, $\:L=1$ represents one such valid link connection and $\:L=0$ represents no link connection.

Constraint 2 indicates that an IoT device transmits information to only one stop position.

Constraint 2: $$\:\sum\:_{j=1}^{S}L=1$$

where, $\:S$ is set of stop position.

Constraint 3: $$\:\sum\:_{i=1}^{t}L\le\:Max$$

Constraint 3 is restricting the number of IoT devices that an UAV can serve is $\:Max$. Variable $\:t$ is the total number of IoT devices.

Constraint 4: $$\:\sum\:_{i=1}^{t}\sum\:_{j=1}^{s}{L}_{ij}=t$$

This constraint verifies that service is covered to all devices. Where $\:t$ is total number of IoT devices.

The proposed Fuzzy based Memetic Algorithm using Diversity control (F-MAD) algorithm is used to solve the above problem and the results attained are assessed using Hyper Volume (HV) metric^67,68^. Since it is a real-world problem and true front for the same is unavailable, HV metric is used as quality indicator. The attained solutions are compared with NSGA-II^39^ and two other algorithms developed to solve above problem.

multiobjective trajectory optimization algorithm with a cutting and padding encoding strategy (MTO-CPE)^65^ and self-adaptive multi-objective differential evolution-based trajectory optimization algorithm (STO)^66^. The attained results are given in Table 12.

**Table 12:** HV metric results.

$\:t$ value (no. of IoT devices)	F-MAD	MTO-CPE	STO
60	**0.524** $\:\pm\:0.00492$	0.510 $\:\pm\:$0.00515	0.5118$\:\pm\:$0.00542
120	**0.428** $\:\pm\:$ **0.00685**	0.378$\:\pm\:$0.00850	0.412$\:\pm\:$0.00784
180	**0.503** $\:\pm\:$ **0.00839**	0.327$\:\pm\:$0.00825	0.487$\:\pm\:$0.00914
300	0.251$\:\pm\:$0.0285	0.243$\:\pm\:0.0148$	**0.278** $\:\pm\:$ **0.0246**
400	0.162$\:\pm\:$0.00479	0.166$\:\pm\:$0.00501	**0.187** $\:\pm\:$ **0.00467**

**Fig. 16:**
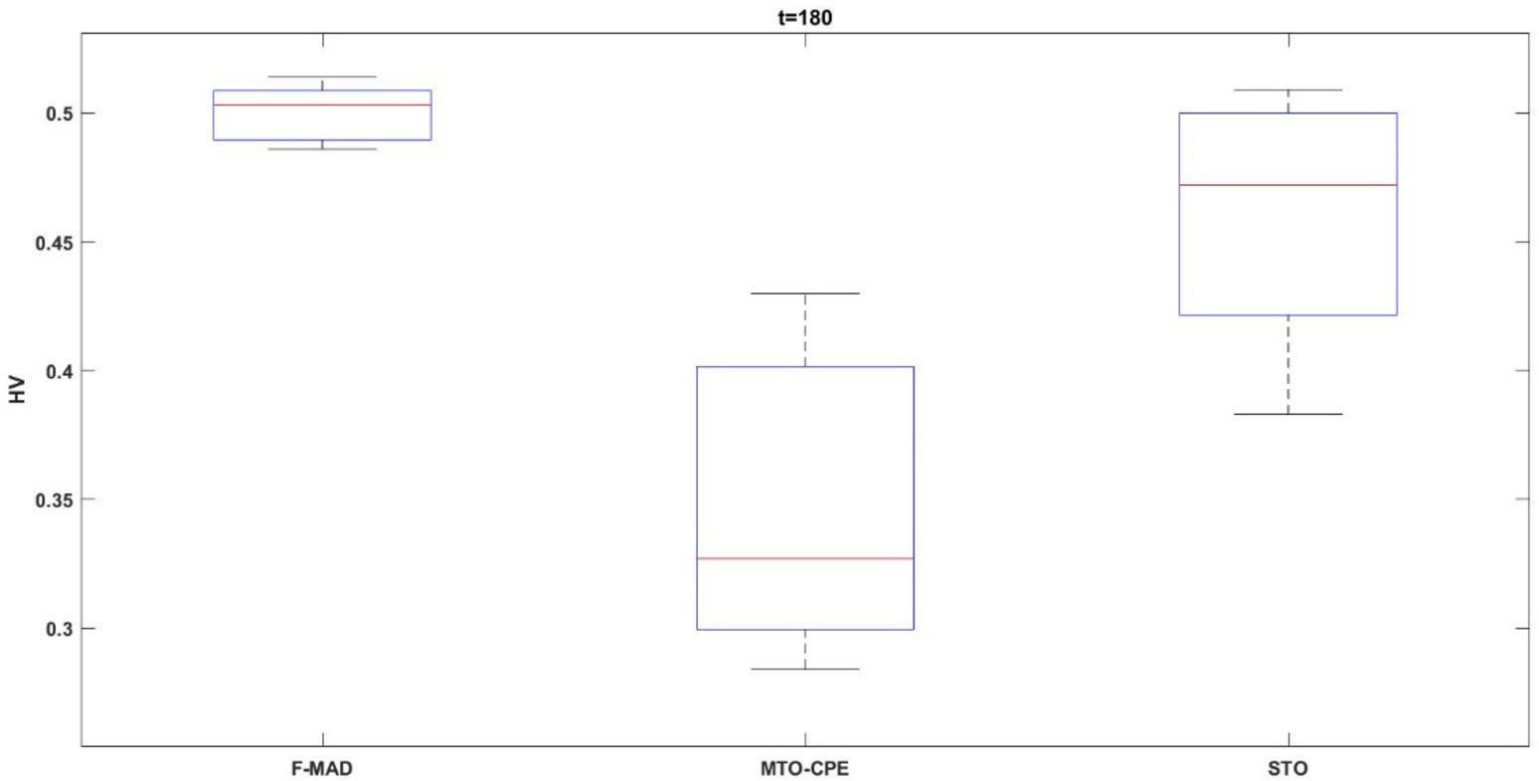
Boxplot HV metric.

The above results show that the proposed F-MAD algorithm attains better HV results for three out of five variants. As the complexity of the $\:t$ variable increases, the algorithms developed and adapted for specific problem exhibit better results. The box-plot for the $\:t=180$ is shown in Fig. 16. From the figure it can be seen that the plot of F-MAD algorithm the solutions are clustered and has less variance, which proves the consistency of the algorithm.

## Conclusions

A Fuzzy based Memetic Algorithm using Diversity Control (F-MAD) is proposed. In the present work, two fuzzy systems are used to adapt vital parameters crossover rate and scaling factor of DE algorithm. A controlled local search procedure is proposed to improve the exploitation characteristics of the proposed algorithm. The adaptation of control parameter values using fuzzy system based on the population diversity enhances the diversity at the decision space and moreover the non-domination based selection procedure and the local search methods improves the convergence and diversity metrics of attained pareto front in the objective space.

For performance analysis a set of 17 benchmark test problems are taken from CEC 2009 report and DTLZ test set. The evaluation is performed based on the Inverted Generational Distance metric and results are compared with wide range of other popular multi-objective optimization algorithms. F-MAD algorithm has attained better IGD value for eight among ten CEC problems while compared to the other representational algorithms. The performance in optimizing all the DTLZ problems considered for experimentation is significant than other algorithms. The results show the dominance of the F-MAD method in comparison to other algorithms by solving most of the problems with lesser IGD values. The results are further tested and proved using Friedman rank statistical test, and the proposed method is ranked first in the test. The fuzzy based self adaptation of control parameters combined with the controlled local search procedure aids in improving the algorithms efficiency. The proposed F-MAD method is thus robust enough to solve varied optimization problems without the necessity for manual parameter fine tuning. The limitation and future research is working and improving algorithm suitable to solve many-objective optimization problems with high dimensions, where maintaining diversity becomes difficult.

## Data Availability

The datasets used and/or analysed during the current study available from the corresponding author on reasonable request.
